# Use of the energy waveform electrocardiogram to detect subclinical left ventricular dysfunction in patients with type 2 diabetes mellitus

**DOI:** 10.1186/s12933-024-02141-1

**Published:** 2024-03-06

**Authors:** Cheng Hwee Soh, Alex G. C. de Sá, Elizabeth Potter, Amera Halabi, David B. Ascher, Thomas H. Marwick

**Affiliations:** 1 Imaging Research Laboratory, Baker Heart and Diabetes Institute, PO Box 6492, Melbourne, VIC 3004 Australia; 2 Baker Department of Cardiometabolic Health, University of Melbourne, Melbourne, Australia; 3 Computational Biology and Clinical Informatics, Baker Heart and Diabetes Institute, Melbourne, Australia; 4 School of Chemistry and Molecular Biosciences, University of Queensland, Brisbane, Australia; 5 Systems and Computational Biology, Bio21 Institute, Parkville, Australia; 6 Menzies Institute for Medical Research, Hobart, Australia

**Keywords:** Diabetes mellitus, Heart failure, Electrocardiogram, Echocardiography

## Abstract

**Background:**

Recent guidelines propose N-terminal pro-B-type natriuretic peptide (NT-proBNP) for recognition of asymptomatic left ventricular (LV) dysfunction (Stage B Heart Failure, SBHF) in type 2 diabetes mellitus (T2DM). Wavelet Transform based signal-processing transforms electrocardiogram (ECG) waveforms into an energy distribution waveform (ew)ECG, providing frequency and energy features that machine learning can use as additional inputs to improve the identification of SBHF. Accordingly, we sought whether machine learning model based on ewECG features was superior to NT-proBNP, as well as a conventional screening tool—the Atherosclerosis Risk in Communities (ARIC) HF risk score, in SBHF screening among patients with T2DM.

**Methods:**

Participants in two clinical trials of SBHF (defined as diastolic dysfunction [DD], reduced global longitudinal strain [GLS ≤ 18%] or LV hypertrophy [LVH]) in T2DM underwent 12-lead ECG with additional ewECG feature and echocardiography. Supervised machine learning was adopted to identify the optimal combination of ewECG extracted features for SBHF screening in 178 participants in one trial and tested in 97 participants in the other trial. The accuracy of the ewECG model in SBHF screening was compared with NT-proBNP and ARIC HF.

**Results:**

SBHF was identified in 128 (72%) participants in the training dataset (median 72 years, 41% female) and 64 (66%) in the validation dataset (median 70 years, 43% female). Fifteen ewECG features showed an area under the curve (AUC) of 0.81 (95% CI 0.787–0.794) in identifying SBHF, significantly better than both NT-proBNP (AUC 0.56, 95% CI 0.44–0.68, *p* < 0.001) and ARIC HF (AUC 0.67, 95%CI 0.56–0.79, *p* = 0.002). ewECG features were also led to robust models screening for DD (AUC 0.74, 95% CI 0.73–0.74), reduced GLS (AUC 0.76, 95% CI 0.73–0.74) and LVH (AUC 0.90, 95% CI 0.88–0.89).

**Conclusions:**

Machine learning based modelling using additional ewECG extracted features are superior to NT-proBNP and ARIC HF in SBHF screening among patients with T2DM, providing an alternative HF screening strategy for asymptomatic patients and potentially act as a guidance tool to determine those who required echocardiogram to confirm diagnosis.

*Trial registration* LEAVE-DM, ACTRN 12619001393145 and Vic-ELF, ACTRN 12617000116325

**Supplementary Information:**

The online version contains supplementary material available at 10.1186/s12933-024-02141-1.

## Background

Heart failure (HF) may be the most common cardiovascular cause of death among patients with type 2 diabetes mellitus (T2DM) [1]. More than 25% of patients with T2DM are reported to have stage B HF (SBHF, also known as asymptomatic LVD) and this is even more prevalent among patients with long-standing diabetes or poor glycemic control [3–5]. HF is a progressive disorder [2], preceded by subclinical left ventricular dysfunction (LVD), detectable with global longitudinal strain (GLS), diastolic indices or LV mass [1]. The reliable detection of SBHF would allow earlier initiation of cardioprotective treatment and prevention or delay of symptomatic HF.

A 2022 consensus report from the American Diabetes Association recommended annual testing of N-terminal pro-B-type natriuretic peptide (NT-proBNP) among patients with T2DM to facilitate early detection of HF [6]. However, the standard cutoff value for NT-proBNP performs better in ruling out patients with no HF than identifying those with HF [7, 8]. This is mainly because comorbidities such as renal diseases, obesity and other cardiovascular diseases (CVD) may also result in the elevation of NT-proBNP value [9]. In our recent experience, neither clinical assessment nor NT-proBNP provided satisfactory discrimination for abnormal GLS (AUC 63%), diastolic indices (e′, AUC 54–61%) or LV mass (AUC 59–67%), and the high sensitivity needed for a screening test was attained only with an unacceptably low (< 50%) specificity [10]. An alternate approach in SBHF screening is the application of the Atherosclerosis Risk in Communities (ARIC) HF risk score, which consists of various risk factors such as age, sex, race, comorbidities and vital signs [11]. It has been previously reported as a strong predictor for HF and yet, the performance in screening for SBHF, an asymptomatic cardiovascular dysfunction, remained contentious.

The energy waveform electrocardiogram (ewECG) uses continuous wavelet transform (CWT) based signal processing to provide time-based Fourier Transform frequency domain information of the acquired ECG signal. This is a feasible screening tool as the 12-lead ECG is a standard test and no additional expertise is required and the ewECG waveform is simply post-acquisition signal processing that takes a few seconds. ewECG extracted features have been shown to be key machine learning model features that improve overall model performance above and beyond conventional ECG features for machine learning models built to be predictive for LV diastolic dysfunction [12, 13] and more recently SBHF in a community population at risk of HF [14]. However, although patients with T2DM have a heightened risk of HF, this is heterogeneous, with systolic as well as diastolic dysfunction being involved [15]. Accordingly, in order to determine an effective way to screen for SBHF and guide clinicians on those who required further echocardiogram, we used machine learning algorithms to develop a DM-specific screening model based on ewECG data to identify SBHF among asymptomatic patients and compare its performance with conventional clinical HF risk indicators in NT-proBNP and the ARIC HF risk score.

## Methods

### Study design and setting

The training dataset comprised asymptomatic participants with T2DM from the *Determining the Effect of Dapagliflozin on Preventing Heart Failure in Patients with Type 2 Diabetes* (LEAVE-DM, ACTRN 12619001393145) trial. These community-based participants were aged ≥ 65 years recruited from Melbourne but excluded those with: (i) moderate or severe valvular heart disease; (ii) history of HF or hypertrophic cardiomyopathy; (iii) currently undergoing therapy with SGLT2 inhibitors or GLP-1 receptor agonist; (iv) systolic blood pressure < 110 mmHg; (v) estimated glomerular filtration rate < 45 mL/min/1.73 m^2^; (vi) baseline New York Heart Association (NYHA) classification > 2 and (vii) oncologic life expectancy < 12 months.

The validation dataset comprised participants with T2DM In the *Victorian Study of Echocardiographic Detection of Left Ventricular Dysfunction* (Vic-ELF, ACTRN 12617000116325). This community-based study recruited participant aged ≥ 65 years with at least one of the following HF risk factors: (i) obesity (body mass index ≥ 30 kg/m^2^), (ii) diagnosed T2DM, or (iii) hypertension (systolic blood pressure ≥ 140 mmHg or on hypertensive medication). Exclusion criteria included LV ejection fraction ≤ 40%, symptomatic HF, known coronary artery disease (CAD), moderate or severe valvular heart disease, renal impairment and symptoms of HF.

### Participant characteristics

Participants’ baseline characteristics such as age, sex, blood pressure, heart rate and body mass index (BMI) are summarised in Table 1. Risk factors for HF such as hypertension, peripheral vascular disease and family history of coronary artery disease (CAD) and HF were also included. In addition, the Charlson Comorbidity Index (CCI) and ARIC HF risk score were calculated to evaluate the severity of comorbidities and 4-year risk of incident symptomatic HF, respectively. Participants’ NT-proBNP levels were also measured.

**Table 1:** Baseline characteristics

	LEAVE-DM (n = 178)	Vic-ELF (n = 97)	*p*
Age, years	72 [69–76]	70 [68–73]	< 0.001
Female (%)	73 (41.0)	42 (43.3)	0.71
CCI, score	5 [4–6]	1 [1, 2]	< 0.001
SBP, mmHg	145 [134–155]	141 [130–150]	0.04
DBP, mmHg	77 [70–85]	85 [78–90]	< 0.001
HR, bpm	71 [64–83]	71 [65–79]	0.63
BMI, kg/m^2^	29.9 [27.3–34.5]	31.0 [27.4–33.9]	0.66
ARIC HF, score	13.8 [8.0–22.8]	9.7 [6.5–14.7]	< 0.001
Obesity (%)	82 (46.6)	58 (59.8)	0.04
Hypertension (%)	126 (70.8)	80 (82.5)	0.03
Type 2 diabetes (%)	178 (100)	97 (100)	–
Peripheral vascular disease (%)	10 (5.7)	1 (1.0)	0.06
Family history of CAD (%)	95 (53.7)	25 (25.8)	< 0.001
Family history of HF (%)	31 (17.6)	4 (4.1)	0.001
NT-proBNP (pg/mL)	93.0 [50.7–228.3]	43.0 [30.3–88.5]	< 0.001
LVMI (g/m^2^)	82.0 [70.8–98.5]	70.4 [56.6–83.6]	< 0.001
Ejection fraction (%)	59.0 [54.0–62.0]	60.1 [56.6–66.0]	0.004
GLS (−%)	17.4 [16.0–19.2]	18.0 [16.7–20.0]	0.005
E/e′	9.5 [7.9–11.5]	8.5 [7.4–10.7]	0.02
LAVI (mL/m^2^)	30.6 [27.1–40.3]	33.4 [28.3–40.4]	0.19
E/A	0.8 [0.7–0.9]	0.8 [0.7–0.9]	0.93
QOL, score	83 [71–93]	80 [70–90]	0.03
RR interval (ms)	873 [750–977]	893 [817–995]	0.064
PR interval (ms)	170 [156–186]	176 [161–188]	0.142
QRS duration (ms)	90 [84–102]	94 [86–104]	0.291
QT duration (ms)	394 [375–422]	400 [380–424]	0.157
QT_c_ interval (ms)	426 [411–440]	425 [411–439]	0.707

Continuous variables are given as median [IQR]

CCI: Charlson Comorbidity Index; SBP: systolic blood pressure; DBP: diastolic blood pressure; HR: heart rate; BMI: body mass index; ARIC HF: Atherosclerosis Risk in Communities Heart Failure score; CAD: coronary artery disease; HF: heart failure; NT-proBNP: N-terminal prohormone of brain natriuretic peptide; LVMI: left ventricular mass index; GLS: global longitudinal strain; LAVI: left atrial volume index; QOL: quality of life

### ewECG

Participants underwent ewECG evaluation using a commercially-available conventional 12-lead ECG device with additional signal processing to provide the ewECG waveform (MyoVista version 2.0, HeartSciences, Southlake, Texas). This ECG signal is deconstructed and presented in an energy scalogram which illustrates the distribution of energy over time and frequency, known as the “energy waveform”. Energy is expressed as coefficients that represent the agreement between wavelet and signal at different scales, rather than discrete energy measurements [13]. Fourier based signal processing is used extensively in medical devices to improve diagnostic performance. An example is the use of Fourier based Doppler signal processing to assess blood flow in echocardiography. A proprietary software generates a total of 643 CWT features at specific points throughout the cardiac cycle. The ewECG interface also provides a standard 12-lead ECG trace and an automated diagnostic interpretation based on the University of Glasgow 12-lead ECG interpretive analysis algorithm, offering both quantitative parameters and qualitative interpretations [16]. These CWT features are combined with conventional measurements to provide 1124 parameters (Additional file 1: Table S1).

### Echocardiography

A transthoracic 2-dimensional and Doppler echocardiographic study was performed among using standard equipment (ACUSON SC2000, Siemens Healthcare USA, Mountain View, California and GE E95 ultrasound device, GE Healthcare, Chicago, Illinois, United States) and transducer (4V1c, 1.25 to 4.5 MHz; 4Z1c, 1.5 to 3.5 MHz) in accordance with the American Society of Echocardiography guidelines. Subclinical LV systolic function was assessed with GLS computed using speckle tracking (Syngo VVI; Siemens Healthcare USA) [17]. Diastolic function was assessed from transmitral flow (peak early [E] and late diastolic velocity E/A ratio), mitral annular early diastolic velocities (e′) and E/e′ ratio, left atrial volume index (LAVi) indexed to body surface area, and tricuspid regurgitation velocity [18].

SBHF was defined by the presence of one or more of: (i) diastolic dysfunction (DD) (E/e′ > 15 or E/e′ > 10 with left atrium enlargement (LAE) or impaired relaxation with LAE); (ii) reduced GLS (≤ 18%); or (iii) left ventricular hypertrophy (LVH, LV mass index > 95 g/m^2^ in women and > 115 g/m^2^ in men).

### Machine learning classification model

A supervised machine learning approach was applied to develop a screening model for the primary outcome—SBHF and three secondary outcomes—(i) DD; (ii) reduced GLS and (iii) LVH. The proposed models for these outcomes included extremely randomised trees [19], random forest [20], explainable boosting machine (EBM) [21], and extreme gradient boosting (XGBoost) [22], all of which were applied using the Scikit-learn [23] and XGBoost and EBM libraries.

Given the high dimensionality of the data from the ewECG features, a bottom-up step-forward feature selection method was performed to identify high-predictive features for the selected outcomes. The feature selection started with zero features in the feature set and added one feature at a time across its iterative process. After including each feature, the feature set was evaluated using a k-fold cross-validation procedure over the training dataset on a machine learning model, where k was set to five based on the size of LEAVE-DM and Vic-ELF cohorts. In summary, k-fold cross-validation assessed the predictive performance of the model by splitting the data into k folds, training and learning it on k– 1 folds, and subsequently validating it on the remaining fold. The evaluation of each feature relied on both sensitivity and AUC of the model. After this step, the best performing feature was combined with the remaining feature set. This iterative process continued until all features were assessed or stopping criteria were achieved (e.g. deterioration or stabilisation of the predictive performance when including new features to the feature set).

The Shapley Additive Explanations (SHAP) method—which provides an overview of how the features are generally linked to the model’s prediction [24]—was then applied to explain the ewECG models. SHAP is a game theory-based method to derive explanations for individual prediction, in which combined results yield a global interpretation of the model’s behavior.

### Statistical analysis

Descriptive statistics were used to present the participants’ baseline characteristics. Parametric variables were reported as means ± standard deviations while non-parametric variables were reported as medians [interquartile ranges (IQR)]. To compare participants’ baseline characteristics between the training and validation dataset, Kruskal–Wallis H test was performed on non-parametric variables and chi-square test on categorical variables.

The performance of each model in identifying the corresponding outcomes (SBHF, DD, reduced GLS and LVH) among the training and validation dataset were reported using the area under the receiver operating characteristics (ROC) curve (AUC). The optimal threshold for the machine learning model was set as 0.5 to identify the model’s sensitivity and specificity. The performance of NT-proBNP and ARIC HF risk score in identifying the outcomes among participants from the validation dataset was also evaluated and compared using the AUC. The optimal cut-off value for NT-proBNP and ARIC HF was identified using Youden’s Index and the accuracy in identifying participants with each outcome was demonstrated.

A *p*-value of < 0.05 was considered statistically significant for all analyses. Analyses were conducted using Stata 17.0 (StataCorp, College Station, Texas, USA) and Python (Python Software Foundation).

## Results

### Patient characteristics

The training (n = 178) and validation (n = 97) datasets comprised elderly patients with T2DM and risk factors, but participants from the training dataset were older, at higher risk of comorbidity and HF, and had a stronger family history of CAD and HF than in the validation dataset (Table 1). Participants from the training dataset had higher NT-proBNP, higher left ventricular mass index, and marginally worse cardiac function. Participants from the training dataset were also shown to have a higher risk of HF incidence given the significantly greater ARIC HF risk score than the validation dataset.

### ewECG machine learning models

**Fig. 1:**
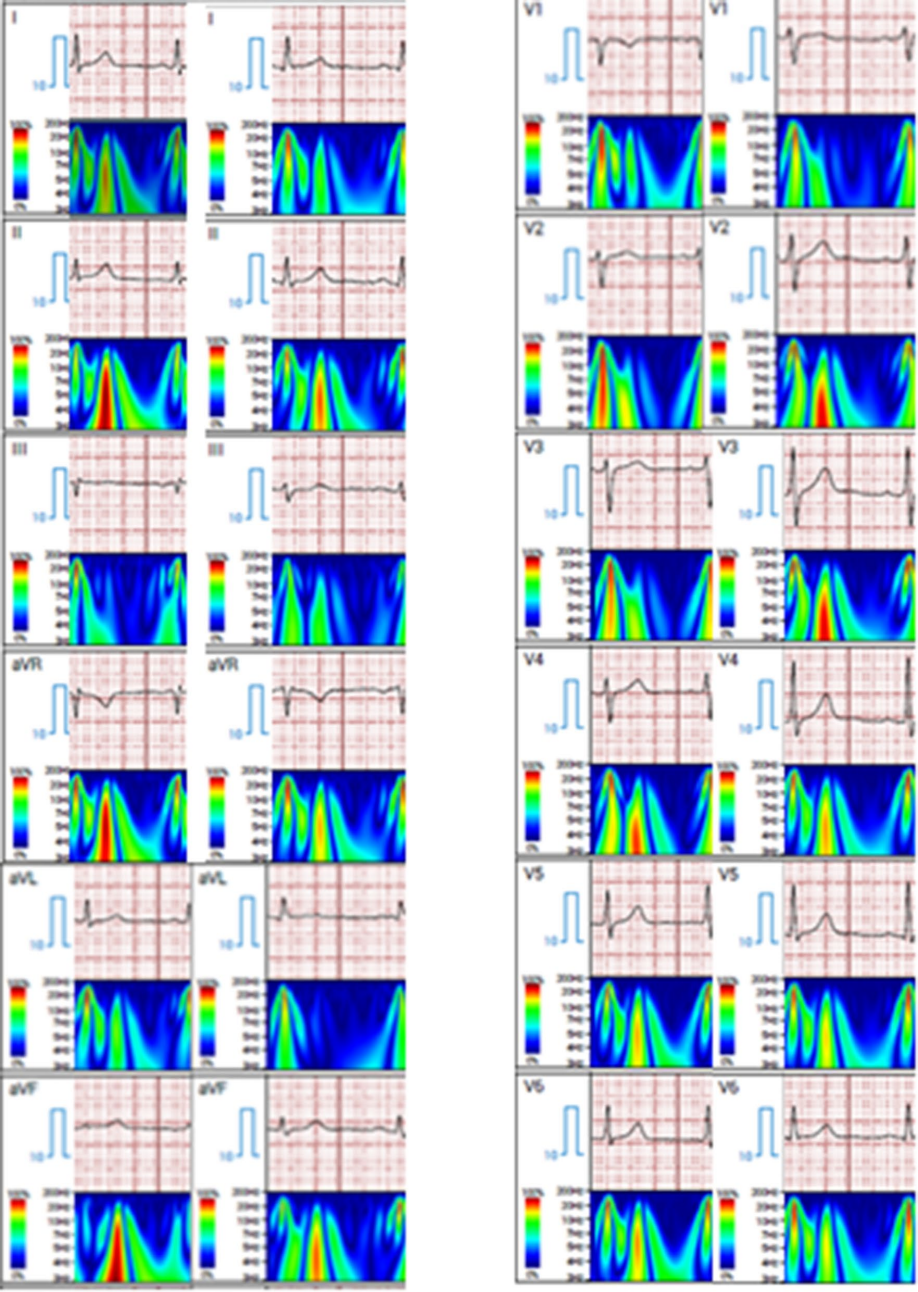
Parametric display of ewECG for patients with and without Stage B Heart Failure. The detection of SBHF was predominantly based on LV systolic markers, emphasized by differences in the energy signal in the parametric display. The color display is referenced to The color displays peak energy relative to the peak of the R-wave (the highest energy component of the waveform)

The ewECGs for T2DM patients with and without SBHF were demonstrated in Additional file 1. From the training dataset, models were developed for the four outcomes based on feature selection from the 643 ewECG features. Additional file 1 provides a detailed description for each of these ewECG features. The features selected in each model were also listed in Additional file 2: Figures. Overall, 15 features were selected with an explainable boosting machine model to identify participants with SBHF (Additional file 2: Fig. S2, n = 128, 71.9%); 11 features for DD (Additional file 2: Fig. S4, n = 66, 37.1%) with an XGBoost model; 18 features for reduced GLS (Additional file 2: Fig. S4, n = 99, 55.6%) with a random forest model and 22 features for LVH (Additional file 2: Fig. S5, n = 36, 20.2%) with an extremely randomised trees model. None of the 15 ewECG features from the SBHF screening model was also selected across all other three outcomes, although there was a preponderance of LV systolic markers, emphasized by differences in the energy signal in the parametric display (Fig. 1). In the training dataset, the SBHF model showed an AUC of 0.87 (95% CI 0.83–0.84), with 98% sensitivity and 58% specificity. Separate models were also developed for other outcomes with different constituents, which led to acceptable discrimination for DD (AUC 0.83 [95% CI 0.81–0.82]), impaired GLS (0.84 [95%CI 0.84–0.85]) and LVH (0.96 [95%CI 0.95–0.952]) in the training dataset.

### NT-pro-BNP and the ARIC HF risk score

The optimal cut-off value for NT-proBNP was identified in the validation dataset using Youden’s Index. Compared with the recommended cut-off value of 125 pg/mL, Youden's Index identified that 21 pg/mL in NT-proBNP showed the highest screening accuracy for SBHF, hence being deemed as the optimal cut-off value. Table 2 demonstrates the AUC for applying NT-proBNP as a screening tool for SBHF, DD, reduced GLS and LVH, as well as the sensitivity and specificity for both recommended (125 pg/mL) and optimal (21 pg/mL) cut-off values. Meanwhile, a score of 8% was deemed as the optimal cut-off value for the ARIC HF risk score for SBHF screening,

**Table 2:** The accuracy of NT-proBNP and ARIC HF in identifying Stage B Heart Failure, diastolic dysfunction, reduced global longitudinal strain and left ventricular hypertrophy in the validation dataset

	NT-proBNP					ARIC HF		
	AUC (95% CI)	> 125 pg/mL (Recommended cut-off)		> 21 pg/mL (optimal cut-off)		AUC (95%CI)	> 8% (optimal cut-off)	
		Sensitivity	Specificity	Sensitivity	Specificity		Sensitivity	Specificity
SBHF	0.55 (0.43–0.66)	14.75%	85.25%	85.25%	9.68%	0.67 (0.56–0.79)	71.88%	66.67%
DD	0.52 (0.40–0.63)	9.76%	87.80%	87.80%	13.73%	0.61 (0.50–0.73)	72.09%	51.85%
Reduced GLS	0.52 (0.41–0.64)	10.87%	84.78%	84.78%	10.87%	0.69 (0.58–0.80)	77.55%	60.42%
LVH	0.66 (0.21–0.98)	60%	80.00%	80.00%	12.64%	0.45 (0.22–0.67)	60%	41.30%

NT-proBNP: N-terminal pro-B-type natriuretic peptide; ARIC HF: atherosclerosis Risk in Communities Heart Failure score; AUC: area under the curve; CI: confidence interval; SBHF: stage B Heart Failure; DD: diastolic dysfunction; GLS: global longitudinal strain; LVH: left ventricular hypertrophy

### Interpretations of ewECG models

The SHAP plot in Additional file 2: Fig. S2 details and ranks the most important CWT features for the SBHF screening model. The amplitude of R-wave in lead II, early repolarization in lead V1 and duration of QRS interval in lead V3 are some of the key features identified in SBHF screening. Additional file 2: Fig. S2 demonstrates that higher R-wave amplitude in lead II and longer duration of QRS interval in lead V3 indicated a greater risk of SBHF. Conversely, a mixed pattern was identified for features such as R-wave amplitude in lead I and T-wave onset in lead II.

Correspondingly, the T-wave area in lead V2 and P-wave amplitude in lead III were key identifiers for DD (Additional file 2: Fig. S3). The amplitude of ST interval in lead II was the most impactful feature in the screening for reduced GLS (Additional file 2: Fig. S4). The frequency of peak depolarization origin measure in lead V3 and delta wave confidence in lead II were some of the key features in the ewECG screening model for LVH (Additional file 2: Fig. S5).

### Comparison of the screening strategies for LV dysfunction

**Fig. 2:**
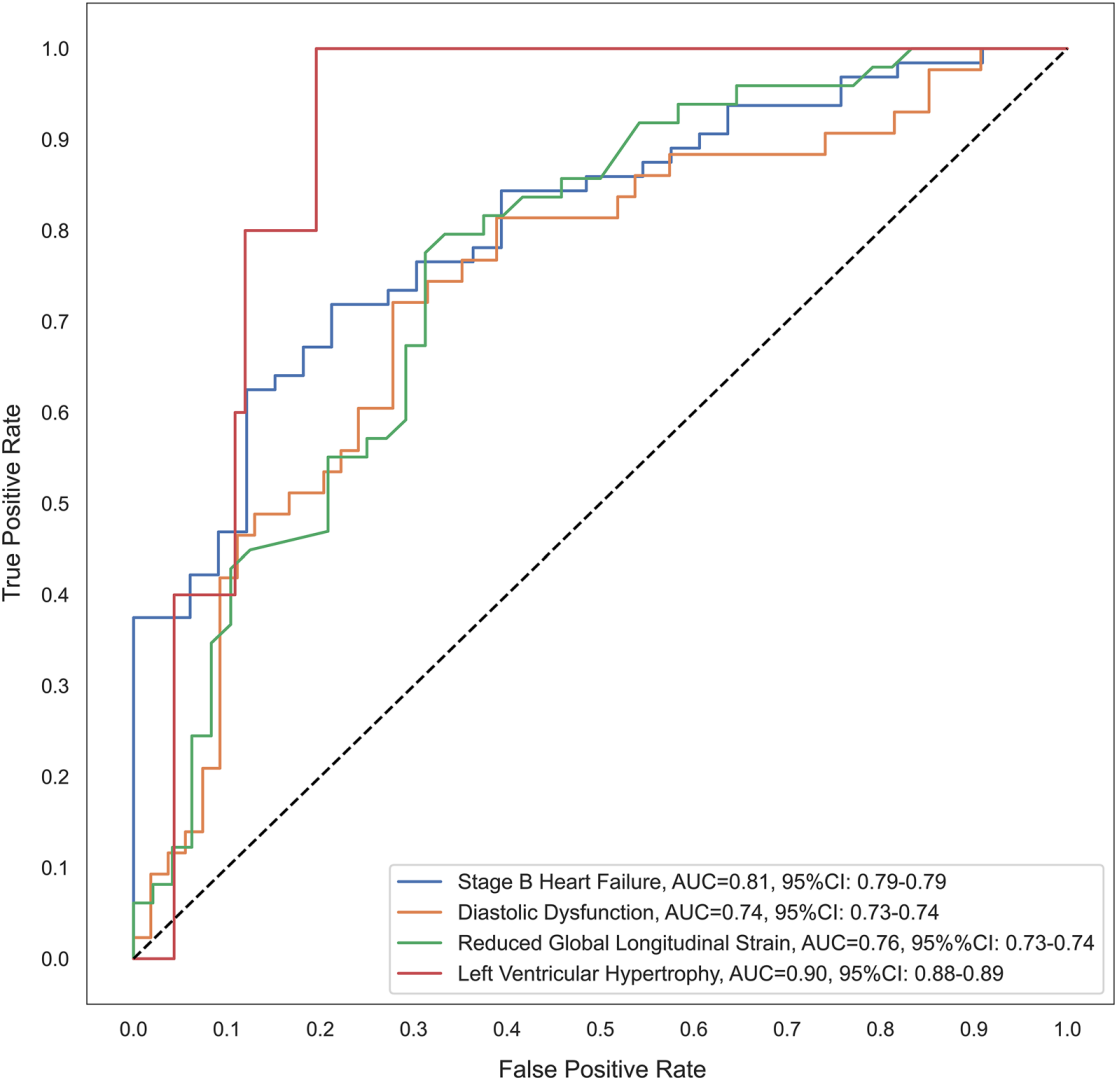
Receiver operating characteristics curve for ewECG models developed to identify Stage B Heart Failure and its components. Similar levels of discrimination were obtained for Stage B Heart Failure, diastolic dysfunction and reduced global longitudinal strain. The curve for left ventricular hypertrophy should be interpreted with caution, because of sparse numbers with this manifestation. ewECG: Electrocardiographic with signal-processed via continuous wavelet transform

A total of 64 participants from the validation dataset (66%) had SBHF. The ewECG screening features were predictive for SBHF in the validation dataset (AUC = 0.81, 95%CI 0.787–0.794), outperforming both NT-proBNP and ARIC HF risk score significantly, with a sensitivity of 89% and specificity of 62% (Fig. 2). At a recommended cut-off value of 125 pg/mL, NT-proBNP was less discriminative for SBHF (AUC 0.55 [95%CI 0.43–0.66]), with a sensitivity of 15% and specificity of 94% (Table 2). On the other hand, applying the optimal cut-off value (21 pg/mL) for NT-proBNP resulted in a sensitivity of 85% and a specificity of 10%. Meanwhile, the ARIC HF yielded an AUC of 0.67 (95%CI 0.56–0.79) in SBHF screening.

**Fig. 3:**
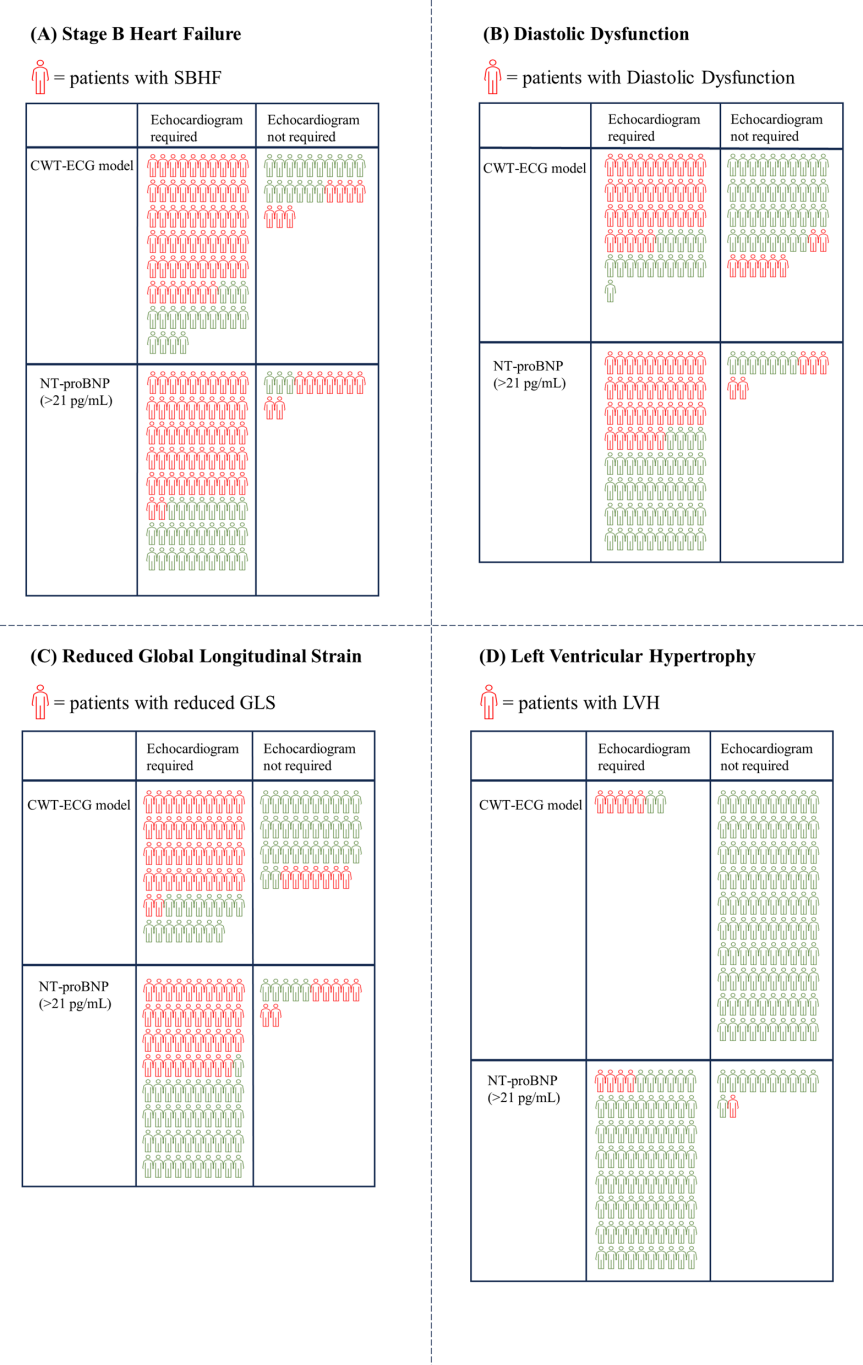
Comparison of screening methods to identify Stage B Heart Failure and its components. This infographic compares the prediction and exclusion of SBHF with ewECG and NT-proBNP. CWT-ECG: Electrocardiographic with signal-processed via continuous wavelet transform; NT-proBNP: N-terminal pro-B-type natriuretic peptide; SBHF: Stage B Heart Failure; GLS: global longitudinal strain; LVH: left ventricular hypertrophy

Figure 3 illustrates the number of participants who would be advised to have echocardiography based on ewECG model versus NT-proBNP as a screening tool for SBHF. The ewECG model identified 67 out of the 97 participants from the validation dataset as participants who required echocardiography, of whom 85% were deemed to have SBHF. In comparison, the application of NT-proBNP, with cutoff values of 125 pg/mL and 21 pg/mL, as a screening tool resulted in unnecessary echocardiography in 85% and 35% of participants respectively.

The prevalence of DD in participants from the validation dataset was 44% (n = 43). The ewECG model (AUC = 0.74, 95% CI 0.73–0.74) outperformed both NT-proBNP-21 pg/mL (AUC = 0.52, 95%CI 0.40–0.63) and ARIC HF (AUC = 0.61, 95%CI 0.50–0.73) in screening for DD. The sensitivities of the ewECG and NT-proBNP-21 pg/mL approaches were similar (81% and 88%, *p* = 0.347), but the specificity of ewECG exceeded that of NT-proBNP (71% vs 14%, *p* < 0.001). As for NT-proBNP with a cut-off value of 125 pg/mL, the sensitivity and specificity were 10% and 86% respectively.

Reduction in GLS (–18% or worse) was present in 51% of participants from the validation dataset (n = 49). Again, the ewECG model showed a higher AUC (0.76, 95%CI 0.73–0.74) in screening for reduced GLS than NT-proBNP-21 pg/mL (AUC = 0.52, 95%CI 0.41–0.64). The ewECG model for reduced GLS accurately screened 76% of the participants, compared to 48% using NT-proBNP. The ARIC HF risk score performed slightly better than NT-proBNP in screening for reduced GLS (AUC = 0.69, 95%CI 0.58–0.80).

Only 5 participants in the validation dataset (5.2%) had LVH. The AUC of the ewECG model was 0.90 (95%CI 0.88–0.89), with a sensitivity of 100% and specificity of 98%. The AUC for NT-proBNP and the ARIC HF risk score were 0.66 (95%CI 0.21–0.98) and 0.45 (95%CI 0.22–0.67) respectively.

## Discussion

Our study demonstrates that machine learning models developed using ewECG features may provide a screening tool to provide clinical guidance for patients with T2DM who may benefit from echocardiography. The ewECG models for SBHF and other abnormalities (DD, reduced GLS and LVH) differed in the features selected, analogous to previous experience in people at risk of HF [14]. The ewECG model for SBHF had an accuracy of 83% in reflecting those who required further echocardiographic screening, compared to 60% with NT-proBNP and 69% with the ARIC HF risk score. These results suggest that ECG measures may be superior to current conventional tool for SBHF screening and that the ewECG could be routinely implemented and monitored among T2DM patients.

### Importance of SBHF diagnoses in the prevention of HF progression

SBHF is relatively common in T2DM, especially in older individuals, those with long-standing diabetes and patients with poor glycemic control [3–5]. SBHF is an independent risk factor for incident HF—the cumulative probability of developing HF within 5 years is 37%—and increases with increasing SBHF severity [25]. Patients with T2DM typically develop HFpEF, and compared to people without T2DM, have a greater degree of LVH, higher circulating markers of oxidative stress, inflammation and fibrosis, and a greater risk of hospitalization [26]. Identification of SBHF among people with T2DM would provide an opportunity to initiate cardioprotective strategies, provide more intense follow-up, and facilitate treatment adherence. Despite being a precursor for subsequent HF incidence, SBHF is still underdiagnosed in T2DM [27]. Not all patients with T2DM are at equal risk, so a clinical score or biomarker would be helpful to identify the patients who need attention.

### CVD screening using ECG assessment

A number of studies have reported the feasibility of applying advanced artificial intelligence (AI) methods to ECG assessment for predicting CVD [27]. AI algorithms such as convolutional neural networks require a training database of ECG interpretation to mimic human-like interpretation of the ECG [29]. ECG interpretation using this approach has been shown to predict low LV ejection fraction (≤ 35%) [30], atrial fibrillation [31], hypertrophic cardiomyopathy [32] and myocardial infarction [33]. Utilizing AI tools to guide clinicians in ECG interpretation can improve patient care by increasing the accuracy of identifying asymptomatic patients who may require further attention. In the context of left ventricular systolic dysfunction, previous study has shown that clinicians who adopted AI-ECG algorithm were twice as likely to diagnose patients with low ejection fraction as those who do not [34]. In addition, the implementation of AI-ECG in systolic dysfunction screening was reported to have an AUC of up to 0.93 [35], which would be highly cost-effective as a guideline for echocardiogram referral if such result is transferable in clinical setting. Nonetheless, AI-ECG approaches perform best in the type of population on which they have been trained, and potential issues with reproducibility may occur due to differences in population characteristics, including racial differences [28].

The potential number of ewECG features is huge, producing a highly-dimensional database for the ewECG assessment process. The sample size required to train the model is significantly smaller than the AI-approach. The predictive performance was generalizable when training and cross-validating these models on LEAVE-DM and independently validating them on the Vic-ELF cohorts. This is highlighted by only a small reduction in predictive performance, based on AUC, sensitivity and specificity, from cross-validation on the training dataset to the validation data.

The introduction of machine learning models based on ewECG as a screening tool provides a new alternative to the echocardiography selection process and yet requires no additional training to conduct the assessment as the acquisition is like a standard ECG. The implementation of a SBHF screening model based on ewECG features would improve selection for echocardiographic testing, while missing a few patients with SBHF.

Our study focuses on patients with T2DM because of a variety of reasons. First and foremost, hyperinsulinaemia-induced hypoglycemia can prolong the QTc interval and decrease T-wave area and amplitude, even among healthy individuals [36]. Furthermore, ECG alterations such as QT dispersion, changes in heart rate variability and ST-T changes may be observed even early in the course of DM [37]. Hence, given that the ECG interpretation would differ among diabetic patients, it raises the need to investigate if the accuracy is similar to what was previously reported of AI-ECG in screening for systolic dysfunction is [35].

### Natriuretic peptides, the ARIC HF risk score, and ewECG in SBHF screening

The feasibility of NT-proBNP makes this an attractive option for annual testing [6]. However, while NT-proBNP is valuable for the recognition of unrecognized HF, it may be less sensitive in the recognition of LV dysfunction that is insufficiently advanced to increase myocardial wall stress [10]. A threshold of 125 pg/mL NT-proBNP is widely used in the diagnosis of acute HF [38], albeit as a “rule-out” test [39]. However, the use of this cut-off for the detection of subclinical HF yields a poor accuracy with high specificity but low sensitivity. In this study, we used the training set to identify a threshold for NT-proBNP (21 pg/mL) that provided a sensitive test. However, this change was at the cost of low specificity, and more patients with normal cardiac function were being falsely deemed as being at risk of SBHF. The result would inflate the numbers of unnecessary echocardiograms [40]. These results confirm our previous observations that question the use of NT-proBNP in the detection of LV dysfunction in T2DM.

In contrast, the ARIC HF risk score showed a slightly better discriminative performance than NT-proBNP. This is presumably due to its multifactorial nature as a screening tool which incorporated key risk factors including but not limited to age, sex, comorbidities and medication history [11]. However, it is important to note that the difficulty of identifying patients with SBHF lies on the condition being asymptomatic, which may result in clinicians overlooking the underlying risk factors presented by the patients. With the optimal cut-off value of 8% for SBHF screening, it may contribute further to clinicians' inability to identify those who have or are at risk of developing SBHF.

Among patients with T2DM, the accuracy of ewECG model in SBHF screening was shown to be significantly higher than both NT-proBNP and the ARIC HF risk score in this study. The ewECG model for SBHF screening was shown to reduce the number of echocardiography performed on patients with regular cardiac function by 64%, compared to NT-proBNP at the cut-off value of 21 pg/mL. In contrast, ewECG may be a sensitive first step to select those who require echocardiography and could ultimately improve the cost-effectiveness of echocardiographic screening for SBHF in T2DM. In addition, the ECG provides a widely-available biomarker that has the potential of identifying myocardial disease at an early stage. Regular ewECG assessment may be a feasible alternative to BNP among patients with T2DM who are already at higher risk of HF.

### Study limitations

Our study has a few limitations. First, this is a cross-sectional study which does not capture the development of SBHF over time. The progressive nature of SBHF may impact the discriminative ability of ewECG, especially among patients in the early stage of SBHF. Second, the sample size for this study is relatively low for machine learning. Further studies with a larger and more diverse patient population are required to define broader implications in clinical settings. Lastly, the number of patients with LVH was very limited in the validation dataset, which is likely to have resulted in the overestimation of the ewECG performance in LVH screening.

## Conclusions

Screening for SBHF, followed by the initiation of cardioprotective treatment, may prevent or delay HF occurrence among high-risk patients with T2DM. Nonetheless, clinical guidelines for referring patients to echocardiographic screening are lacking [2]. As ECG assessment is a routine clinical procedure, the application of ewECG is feasible and could act as a tool to identify asymptomatic T2DM patients who require echocardiography for SBHF diagnosis. Future studies should look into whether the findings from our study is consistent in a larger cohort of T2DM patients and investigate the cost-effectiveness of integrating ewECG in clinical settings for SBHF screening.

### Supplementary Information

**Additional file 1.**

**Additional file 2.**

## Data Availability

The datasets used and analysed in this study are available from the corresponding author on reasonable request.

## Abbreviations

ARIC: Atherosclerosis risk in communities
CAD: Coronary artery disease
CWT: Continuous wave transform
ECG: Electrocardiogram
ewECG: Energy waveform electrocardiogram
GLS: Global longitudinal strain
HF: Heart failure
LV: Left ventricle
LVH: Left ventricle hypertrophy
NT-proBNP: N-terminal pro-B-type natriuretic peptide
SBHF: Stage B heart failure
T2DM: Type 2 diabetes mellitus
