# Supplementary material for: Use of the energy waveform electrocardiogram to detect subclinical left ventricular dysfunction in patients with type 2 diabetes mellitus
Source: Cardiovasc Diabetol. 2024 Mar 6;23:91. doi: 10.1186/s12933-024-02141-1 (PMC10918872; doi:10.1186/s12933-024-02141-1)
Supplement: Supplementary file 1 — Additional file 1. [file 12933_2024_2141_MOESM1_ESM.pdf]

ewECG Features	Data Type	Continuous or Categorical	Units	Description
I.DOM	Integer	Continuous	Ratio	CWT: Depolarization Origin (Q) Measure
II.DOM	Integer	Continuous	Ratio	CWT: Depolarization Origin (Q) Measure
III.DOM	Integer	Continuous	Ratio	CWT: Depolarization Origin (Q) Measure
aVR.DOM	Integer	Continuous	Ratio	CWT: Depolarization Origin (Q) Measure
aVL.DOM	Integer	Continuous	Ratio	CWT: Depolarization Origin (Q) Measure
aVF.DOM	Integer	Continuous	Ratio	CWT: Depolarization Origin (Q) Measure
V1.DOM	Integer	Continuous	Ratio	CWT: Depolarization Origin (Q) Measure
V2.DOM	Integer	Continuous	Ratio	CWT: Depolarization Origin (Q) Measure
V3.DOM	Integer	Continuous	Ratio	CWT: Depolarization Origin (Q) Measure
V4.DOM	Integer	Continuous	Ratio	CWT: Depolarization Origin (Q) Measure
V5.DOM	Integer	Continuous	Ratio	CWT: Depolarization Origin (Q) Measure
V6.DOM	Integer	Continuous	Ratio	CWT: Depolarization Origin (Q) Measure
I.freq_DOM	Integer	Continuous	Freq. ID	CWT: Frequency ID at which peak DOM measure was found.
II.freq_DOM	Integer	Continuous	Freq. ID	CWT: Frequency ID at which peak DOM measure was found.
III.freq_DOM	Integer	Continuous	Freq. ID	CWT: Frequency ID at which peak DOM measure was found.
aVR.freq_DOM	Integer	Continuous	Freq. ID	CWT: Frequency ID at which peak DOM measure was found.
aVL.freq_DOM	Integer	Continuous	Freq. ID	CWT: Frequency ID at which peak DOM measure was found.
aVF.freq_DOM	Integer	Continuous	Freq. ID	CWT: Frequency ID at which peak DOM measure was found.
V1.freq_DOM	Integer	Continuous	Freq. ID	CWT: Frequency ID at which peak DOM measure was found.
V2.freq_DOM	Integer	Continuous	Freq. ID	CWT: Frequency ID at which peak DOM measure was found.
V3.freq_DOM	Integer	Continuous	Freq. ID	CWT: Frequency ID at which peak DOM measure was found.
V4.freq_DOM	Integer	Continuous	Freq. ID	CWT: Frequency ID at which peak DOM measure was found.
V5.freq_DOM	Integer	Continuous	Freq. ID	CWT: Frequency ID at which peak DOM measure was found.
V6.freq_DOM	Integer	Continuous	Freq. ID	CWT: Frequency ID at which peak DOM measure was found.
I.DPM	Integer	Continuous	Ratio	CWT: Depolarization Peak (R) Measure
II.DPM	Integer	Continuous	Ratio	CWT: Depolarization Peak (R) Measure
III.DPM	Integer	Continuous	Ratio	CWT: Depolarization Peak (R) Measure
aVR.DPM	Integer	Continuous	Ratio	CWT: Depolarization Peak (R) Measure
aVL.DPM	Integer	Continuous	Ratio	CWT: Depolarization Peak (R) Measure
aVF.DPM	Integer	Continuous	Ratio	CWT: Depolarization Peak (R) Measure
V1.DPM	Integer	Continuous	Ratio	CWT: Depolarization Peak (R) Measure
V2.DPM	Integer	Continuous	Ratio	CWT: Depolarization Peak (R) Measure
V3.DPM	Integer	Continuous	Ratio	CWT: Depolarization Peak (R) Measure
V4.DPM	Integer	Continuous	Ratio	CWT: Depolarization Peak (R) Measure
V5.DPM	Integer	Continuous	Ratio	CWT: Depolarization Peak (R) Measure
V6.DPM	Integer	Continuous	Ratio	CWT: Depolarization Peak (R) Measure
I.freq_DPM	Integer	Continuous	Freq. ID	CWT: Frequency ID at which peak DPM measure was found.
II.freq_DPM	Integer	Continuous	Freq. ID	CWT: Frequency ID at which peak DPM measure was found.
III.freq_DPM	Integer	Continuous	Freq. ID	CWT: Frequency ID at which peak DPM measure was found.
aVR.freq_DPM	Integer	Continuous	Freq. ID	CWT: Frequency ID at which peak DPM measure was found.
aVL.freq_DPM	Integer	Continuous	Freq. ID	CWT: Frequency ID at which peak DPM measure was found.
aVF.freq_DPM	Integer	Continuous	Freq. ID	CWT: Frequency ID at which peak DPM measure was found.
V1.freq_DPM	Integer	Continuous	Freq. ID	CWT: Frequency ID at which peak DPM measure was found.
V2.freq_DPM	Integer	Continuous	Freq. ID	CWT: Frequency ID at which peak DPM measure was found.
V3.freq_DPM	Integer	Continuous	Freq. ID	CWT: Frequency ID at which peak DPM measure was found.
V4.freq_DPM	Integer	Continuous	Freq. ID	CWT: Frequency ID at which peak DPM measure was found.
V5.freq_DPM	Integer	Continuous	Freq. ID	CWT: Frequency ID at which peak DPM measure was found.
V6.freq_DPM	Integer	Continuous	Freq. ID	CWT: Frequency ID at which peak DPM measure was found.

I.DTM	Integer	Continuous	Ratio	CWT: Depolarization Terminal (S) Measure
II.DTM	Integer	Continuous	Ratio	CWT: Depolarization Terminal (S) Measure
III.DTM	Integer	Continuous	Ratio	CWT: Depolarization Terminal (S) Measure
aVR.DTM	Integer	Continuous	Ratio	CWT: Depolarization Terminal (S) Measure
aVL.DTM	Integer	Continuous	Ratio	CWT: Depolarization Terminal (S) Measure
aVF.DTM	Integer	Continuous	Ratio	CWT: Depolarization Terminal (S) Measure
V1.DTM	Integer	Continuous	Ratio	CWT: Depolarization Terminal (S) Measure
V2.DTM	Integer	Continuous	Ratio	CWT: Depolarization Terminal (S) Measure
V3.DTM	Integer	Continuous	Ratio	CWT: Depolarization Terminal (S) Measure
V4.DTM	Integer	Continuous	Ratio	CWT: Depolarization Terminal (S) Measure
V5.DTM	Integer	Continuous	Ratio	CWT: Depolarization Terminal (S) Measure
V6.DTM	Integer	Continuous	Ratio	CWT: Depolarization Terminal (S) Measure
I.freq_DTM	Integer	Continuous	Freq. ID	CWT: Frequency ID at which peak DTM measure was found.
II.freq_DTM	Integer	Continuous	Freq. ID	CWT: Frequency ID at which peak DTM measure was found.
III.freq_DTM	Integer	Continuous	Freq. ID	CWT: Frequency ID at which peak DTM measure was found.
aVR.freq_DTM	Integer	Continuous	Freq. ID	CWT: Frequency ID at which peak DTM measure was found.
aVL.freq_DTM	Integer	Continuous	Freq. ID	CWT: Frequency ID at which peak DTM measure was found.
aVF.freq_DTM	Integer	Continuous	Freq. ID	CWT: Frequency ID at which peak DTM measure was found.
V1.freq_DTM	Integer	Continuous	Freq. ID	CWT: Frequency ID at which peak DTM measure was found.
V2.freq_DTM	Integer	Continuous	Freq. ID	CWT: Frequency ID at which peak DTM measure was found.
V3.freq_DTM	Integer	Continuous	Freq. ID	CWT: Frequency ID at which peak DTM measure was found.
V4.freq_DTM	Integer	Continuous	Freq. ID	CWT: Frequency ID at which peak DTM measure was found.
V5.freq_DTM	Integer	Continuous	Freq. ID	CWT: Frequency ID at which peak DTM measure was found.
V6.freq_DTM	Integer	Continuous	Freq. ID	CWT: Frequency ID at which peak DTM measure was found.
I.REM	Integer	Continuous	Ratio	CWT: Repolarization Early (T) Measure
II.REM	Integer	Continuous	Ratio	CWT: Repolarization Early (T) Measure
III.REM	Integer	Continuous	Ratio	CWT: Repolarization Early (T) Measure
aVR.REM	Integer	Continuous	Ratio	CWT: Repolarization Early (T) Measure
aVL.REM	Integer	Continuous	Ratio	CWT: Repolarization Early (T) Measure
aVF.REM	Integer	Continuous	Ratio	CWT: Repolarization Early (T) Measure
V1.REM	Integer	Continuous	Ratio	CWT: Repolarization Early (T) Measure
V2.REM	Integer	Continuous	Ratio	CWT: Repolarization Early (T) Measure
V3.REM	Integer	Continuous	Ratio	CWT: Repolarization Early (T) Measure
V4.REM	Integer	Continuous	Ratio	CWT: Repolarization Early (T) Measure
V5.REM	Integer	Continuous	Ratio	CWT: Repolarization Early (T) Measure
V6.REM	Integer	Continuous	Ratio	CWT: Repolarization Early (T) Measure
I.freq_REM	Integer	Continuous	Freq. ID	CWT: Frequency ID at which peak REM measure was found.
II.freq_REM	Integer	Continuous	Freq. ID	CWT: Frequency ID at which peak REM measure was found.
III.freq_REM	Integer	Continuous	Freq. ID	CWT: Frequency ID at which peak REM measure was found.
aVR.freq_REM	Integer	Continuous	Freq. ID	CWT: Frequency ID at which peak REM measure was found.
aVL.freq_REM	Integer	Continuous	Freq. ID	CWT: Frequency ID at which peak REM measure was found.
aVF.freq_REM	Integer	Continuous	Freq. ID	CWT: Frequency ID at which peak REM measure was found.
V1.freq_REM	Integer	Continuous	Freq. ID	CWT: Frequency ID at which peak REM measure was found.
V2.freq_REM	Integer	Continuous	Freq. ID	CWT: Frequency ID at which peak REM measure was found.
V3.freq_REM	Integer	Continuous	Freq. ID	CWT: Frequency ID at which peak REM measure was found.
V4.freq_REM	Integer	Continuous	Freq. ID	CWT: Frequency ID at which peak REM measure was found.
V5.freq_REM	Integer	Continuous	Freq. ID	CWT: Frequency ID at which peak REM measure was found.
V6.freq_REM	Integer	Continuous	Freq. ID	CWT: Frequency ID at which peak REM measure was found.
I.RLM	Integer	Continuous	Ratio	CWT: Repolarization Late (T) Measure

II.RLM	Integer	Continuous	Ratio	CWT: Repolarization Late (T) Measure
III.RLM	Integer	Continuous	Ratio	CWT: Repolarization Late (T) Measure
aVR.RLM	Integer	Continuous	Ratio	CWT: Repolarization Late (T) Measure
aVL.RLM	Integer	Continuous	Ratio	CWT: Repolarization Late (T) Measure
aVF.RLM	Integer	Continuous	Ratio	CWT: Repolarization Late (T) Measure
V1.RLM	Integer	Continuous	Ratio	CWT: Repolarization Late (T) Measure
V2.RLM	Integer	Continuous	Ratio	CWT: Repolarization Late (T) Measure
V3.RLM	Integer	Continuous	Ratio	CWT: Repolarization Late (T) Measure
V4.RLM	Integer	Continuous	Ratio	CWT: Repolarization Late (T) Measure
V5.RLM	Integer	Continuous	Ratio	CWT: Repolarization Late (T) Measure
V6.RLM	Integer	Continuous	Ratio	CWT: Repolarization Late (T) Measure
I.freq_RLM	Integer	Continuous	Freq. ID	CWT: Frequency ID at which peak RLM measure was found.
II.freq_RLM	Integer	Continuous	Freq. ID	CWT: Frequency ID at which peak RLM measure was found.
III.freq_RLM	Integer	Continuous	Freq. ID	CWT: Frequency ID at which peak RLM measure was found.
aVR.freq_RLM	Integer	Continuous	Freq. ID	CWT: Frequency ID at which peak RLM measure was found.
aVL.freq_RLM	Integer	Continuous	Freq. ID	CWT: Frequency ID at which peak RLM measure was found.
aVF.freq_RLM	Integer	Continuous	Freq. ID	CWT: Frequency ID at which peak RLM measure was found.
V1.freq_RLM	Integer	Continuous	Freq. ID	CWT: Frequency ID at which peak RLM measure was found.
V2.freq_RLM	Integer	Continuous	Freq. ID	CWT: Frequency ID at which peak RLM measure was found.
V3.freq_RLM	Integer	Continuous	Freq. ID	CWT: Frequency ID at which peak RLM measure was found.
V4.freq_RLM	Integer	Continuous	Freq. ID	CWT: Frequency ID at which peak RLM measure was found.
V5.freq_RLM	Integer	Continuous	Freq. ID	CWT: Frequency ID at which peak RLM measure was found.
V6.freq_RLM	Integer	Continuous	Freq. ID	CWT: Frequency ID at which peak RLM measure was found.
I.REM2	Integer	Continuous	Ratio	CWT: if Rsign=1, REA, else REI
II.REM2	Integer	Continuous	Ratio	CWT: if Rsign=1, REA, else REI
III.REM2	Integer	Continuous	Ratio	CWT: if Rsign=1, REA, else REI
aVR.REM2	Integer	Continuous	Ratio	CWT: if Rsign=1, REA, else REI
aVL.REM2	Integer	Continuous	Ratio	CWT: if Rsign=1, REA, else REI
aVF.REM2	Integer	Continuous	Ratio	CWT: if Rsign=1, REA, else REI
V1.REM2	Integer	Continuous	Ratio	CWT: if Rsign=1, REA, else REI
V2.REM2	Integer	Continuous	Ratio	CWT: if Rsign=1, REA, else REI
V3.REM2	Integer	Continuous	Ratio	CWT: if Rsign=1, REA, else REI
V4.REM2	Integer	Continuous	Ratio	CWT: if Rsign=1, REA, else REI
V5.REM2	Integer	Continuous	Ratio	CWT: if Rsign=1, REA, else REI
V6.REM2	Integer	Continuous	Ratio	CWT: if Rsign=1, REA, else REI
I.freq_REM2	Integer	Continuous	Freq. ID	CWT: Frequency ID at which peak REM2 measure was found.
II.freq_REM2	Integer	Continuous	Freq. ID	CWT: Frequency ID at which peak REM2 measure was found.
III.freq_REM2	Integer	Continuous	Freq. ID	CWT: Frequency ID at which peak REM2 measure was found.
aVR.freq_REM2	Integer	Continuous	Freq. ID	CWT: Frequency ID at which peak REM2 measure was found.
aVL.freq_REM2	Integer	Continuous	Freq. ID	CWT: Frequency ID at which peak REM2 measure was found.
aVF.freq_REM2	Integer	Continuous	Freq. ID	CWT: Frequency ID at which peak REM2 measure was found.
V1.freq_REM2	Integer	Continuous	Freq. ID	CWT: Frequency ID at which peak REM2 measure was found.
V2.freq_REM2	Integer	Continuous	Freq. ID	CWT: Frequency ID at which peak REM2 measure was found.
V3.freq_REM2	Integer	Continuous	Freq. ID	CWT: Frequency ID at which peak REM2 measure was found.
V4.freq_REM2	Integer	Continuous	Freq. ID	CWT: Frequency ID at which peak REM2 measure was found.
V5.freq_REM2	Integer	Continuous	Freq. ID	CWT: Frequency ID at which peak REM2 measure was found.
V6.freq_REM2	Integer	Continuous	Freq. ID	CWT: Frequency ID at which peak REM2 measure was found.
I.RLM2	Integer	Continuous	Ratio	CWT: if Rsign=1, RLI, else RLA
II.RLM2	Integer	Continuous	Ratio	CWT: if Rsign=1, RLI, else RLA

III.RLM2	Integer	Continuous	Ratio	CWT: if Rsign=1, RLI, else RLA
aVR.RLM2	Integer	Continuous	Ratio	CWT: if Rsign=1, RLI, else RLA
aVL.RLM2	Integer	Continuous	Ratio	CWT: if Rsign=1, RLI, else RLA
aVF.RLM2	Integer	Continuous	Ratio	CWT: if Rsign=1, RLI, else RLA
V1.RLM2	Integer	Continuous	Ratio	CWT: if Rsign=1, RLI, else RLA
V2.RLM2	Integer	Continuous	Ratio	CWT: if Rsign=1, RLI, else RLA
V3.RLM2	Integer	Continuous	Ratio	CWT: if Rsign=1, RLI, else RLA
V4.RLM2	Integer	Continuous	Ratio	CWT: if Rsign=1, RLI, else RLA
V5.RLM2	Integer	Continuous	Ratio	CWT: if Rsign=1, RLI, else RLA
V6.RLM2	Integer	Continuous	Ratio	CWT: if Rsign=1, RLI, else RLA
I.freq_RLM2	Integer	Continuous	Freq. ID	CWT: Frequency ID at which peak RLM2 measure was found.
II.freq_RLM2	Integer	Continuous	Freq. ID	CWT: Frequency ID at which peak RLM2 measure was found.
III.freq_RLM2	Integer	Continuous	Freq. ID	CWT: Frequency ID at which peak RLM2 measure was found.
aVR.freq_RLM2	Integer	Continuous	Freq. ID	CWT: Frequency ID at which peak RLM2 measure was found.
aVL.freq_RLM2	Integer	Continuous	Freq. ID	CWT: Frequency ID at which peak RLM2 measure was found.
aVF.freq_RLM2	Integer	Continuous	Freq. ID	CWT: Frequency ID at which peak RLM2 measure was found.
V1.freq_RLM2	Integer	Continuous	Freq. ID	CWT: Frequency ID at which peak RLM2 measure was found.
V2.freq_RLM2	Integer	Continuous	Freq. ID	CWT: Frequency ID at which peak RLM2 measure was found.
V3.freq_RLM2	Integer	Continuous	Freq. ID	CWT: Frequency ID at which peak RLM2 measure was found.
V4.freq_RLM2	Integer	Continuous	Freq. ID	CWT: Frequency ID at which peak RLM2 measure was found.
V5.freq_RLM2	Integer	Continuous	Freq. ID	CWT: Frequency ID at which peak RLM2 measure was found.
V6.freq_RLM2	Integer	Continuous	Freq. ID	CWT: Frequency ID at which peak RLM2 measure was found.
I.REI	Integer	Continuous	Ratio	CWT: Repolarization Early mInimum
II.REI	Integer	Continuous	Ratio	CWT: Repolarization Early mInimum
III.REI	Integer	Continuous	Ratio	CWT: Repolarization Early mInimum
aVR.REI	Integer	Continuous	Ratio	CWT: Repolarization Early mInimum
aVL.REI	Integer	Continuous	Ratio	CWT: Repolarization Early mInimum
aVF.REI	Integer	Continuous	Ratio	CWT: Repolarization Early mInimum
V1.REI	Integer	Continuous	Ratio	CWT: Repolarization Early mInimum
V2.REI	Integer	Continuous	Ratio	CWT: Repolarization Early mInimum
V3.REI	Integer	Continuous	Ratio	CWT: Repolarization Early mInimum
V4.REI	Integer	Continuous	Ratio	CWT: Repolarization Early mInimum
V5.REI	Integer	Continuous	Ratio	CWT: Repolarization Early mInimum
V6.REI	Integer	Continuous	Ratio	CWT: Repolarization Early mInimum
I.freq_REI	Integer	Continuous	Freq. ID	CWT: Frequency ID at which peak REI measure was found.
II.freq_REI	Integer	Continuous	Freq. ID	CWT: Frequency ID at which peak REI measure was found.
III.freq_REI	Integer	Continuous	Freq. ID	CWT: Frequency ID at which peak REI measure was found.
aVR.freq_REI	Integer	Continuous	Freq. ID	CWT: Frequency ID at which peak REI measure was found.
aVL.freq_REI	Integer	Continuous	Freq. ID	CWT: Frequency ID at which peak REI measure was found.
aVF.freq_REI	Integer	Continuous	Freq. ID	CWT: Frequency ID at which peak REI measure was found.
V1.freq_REI	Integer	Continuous	Freq. ID	CWT: Frequency ID at which peak REI measure was found.
V2.freq_REI	Integer	Continuous	Freq. ID	CWT: Frequency ID at which peak REI measure was found.
V3.freq_REI	Integer	Continuous	Freq. ID	CWT: Frequency ID at which peak REI measure was found.
V4.freq_REI	Integer	Continuous	Freq. ID	CWT: Frequency ID at which peak REI measure was found.
V5.freq_REI	Integer	Continuous	Freq. ID	CWT: Frequency ID at which peak REI measure was found.
V6.freq_REI	Integer	Continuous	Freq. ID	CWT: Frequency ID at which peak REI measure was found.
I.REA	Integer	Continuous	Ratio	CWT: Repolarization Early mAximum
II.REA	Integer	Continuous	Ratio	CWT: Repolarization Early mAximum
III.REA	Integer	Continuous	Ratio	CWT: Repolarization Early mAximum

aVR.REA	Integer	Continuous	Ratio	CWT: Repolarization Early mAximum
aVL.REA	Integer	Continuous	Ratio	CWT: Repolarization Early mAximum
aVF.REA	Integer	Continuous	Ratio	CWT: Repolarization Early mAximum
V1.REA	Integer	Continuous	Ratio	CWT: Repolarization Early mAximum
V2.REA	Integer	Continuous	Ratio	CWT: Repolarization Early mAximum
V3.REA	Integer	Continuous	Ratio	CWT: Repolarization Early mAximum
V4.REA	Integer	Continuous	Ratio	CWT: Repolarization Early mAximum
V5.REA	Integer	Continuous	Ratio	CWT: Repolarization Early mAximum
V6.REA	Integer	Continuous	Ratio	CWT: Repolarization Early mAximum
I.freq_REA	Integer	Continuous	Freq. ID	CWT: Frequency ID at which peak REA measure was found.
II.freq_REA	Integer	Continuous	Freq. ID	CWT: Frequency ID at which peak REA measure was found.
III.freq_REA	Integer	Continuous	Freq. ID	CWT: Frequency ID at which peak REA measure was found.
aVR.freq_REA	Integer	Continuous	Freq. ID	CWT: Frequency ID at which peak REA measure was found.
aVL.freq_REA	Integer	Continuous	Freq. ID	CWT: Frequency ID at which peak REA measure was found.
aVF.freq_REA	Integer	Continuous	Freq. ID	CWT: Frequency ID at which peak REA measure was found.
V1.freq_REA	Integer	Continuous	Freq. ID	CWT: Frequency ID at which peak REA measure was found.
V2.freq_REA	Integer	Continuous	Freq. ID	CWT: Frequency ID at which peak REA measure was found.
V3.freq_REA	Integer	Continuous	Freq. ID	CWT: Frequency ID at which peak REA measure was found.
V4.freq_REA	Integer	Continuous	Freq. ID	CWT: Frequency ID at which peak REA measure was found.
V5.freq_REA	Integer	Continuous	Freq. ID	CWT: Frequency ID at which peak REA measure was found.
V6.freq_REA	Integer	Continuous	Freq. ID	CWT: Frequency ID at which peak REA measure was found.
I.RPM	Integer	Continuous	Ratio	CWT: Repolarization Peak (T) Measure
II.RPM	Integer	Continuous	Ratio	CWT: Repolarization Peak (T) Measure
III.RPM	Integer	Continuous	Ratio	CWT: Repolarization Peak (T) Measure
aVR.RPM	Integer	Continuous	Ratio	CWT: Repolarization Peak (T) Measure
aVL.RPM	Integer	Continuous	Ratio	CWT: Repolarization Peak (T) Measure
aVF.RPM	Integer	Continuous	Ratio	CWT: Repolarization Peak (T) Measure
V1.RPM	Integer	Continuous	Ratio	CWT: Repolarization Peak (T) Measure
V2.RPM	Integer	Continuous	Ratio	CWT: Repolarization Peak (T) Measure
V3.RPM	Integer	Continuous	Ratio	CWT: Repolarization Peak (T) Measure
V4.RPM	Integer	Continuous	Ratio	CWT: Repolarization Peak (T) Measure
V5.RPM	Integer	Continuous	Ratio	CWT: Repolarization Peak (T) Measure
V6.RPM	Integer	Continuous	Ratio	CWT: Repolarization Peak (T) Measure
I.freq_RPM	Integer	Continuous	Freq. ID	CWT: Frequency ID at which peak RPM measure was found.
II.freq_RPM	Integer	Continuous	Freq. ID	CWT: Frequency ID at which peak RPM measure was found.
III.freq_RPM	Integer	Continuous	Freq. ID	CWT: Frequency ID at which peak RPM measure was found.
aVR.freq_RPM	Integer	Continuous	Freq. ID	CWT: Frequency ID at which peak RPM measure was found.
aVL.freq_RPM	Integer	Continuous	Freq. ID	CWT: Frequency ID at which peak RPM measure was found.
aVF.freq_RPM	Integer	Continuous	Freq. ID	CWT: Frequency ID at which peak RPM measure was found.
V1.freq_RPM	Integer	Continuous	Freq. ID	CWT: Frequency ID at which peak RPM measure was found.
V2.freq_RPM	Integer	Continuous	Freq. ID	CWT: Frequency ID at which peak RPM measure was found.
V3.freq_RPM	Integer	Continuous	Freq. ID	CWT: Frequency ID at which peak RPM measure was found.
V4.freq_RPM	Integer	Continuous	Freq. ID	CWT: Frequency ID at which peak RPM measure was found.
V5.freq_RPM	Integer	Continuous	Freq. ID	CWT: Frequency ID at which peak RPM measure was found.
V6.freq_RPM	Integer	Continuous	Freq. ID	CWT: Frequency ID at which peak RPM measure was found.
I.RPI	Integer	Continuous	Ratio	CWT: Repolarization Peak mInimum
II.RPI	Integer	Continuous	Ratio	CWT: Repolarization Peak mInimum
III.RPI	Integer	Continuous	Ratio	CWT: Repolarization Peak mInimum
aVR.RPI	Integer	Continuous	Ratio	CWT: Repolarization Peak mInimum

aVL.RPI	Integer	Continuous	Ratio	CWT: Repolarization Peak mInimum
aVF.RPI	Integer	Continuous	Ratio	CWT: Repolarization Peak mInimum
V1.RPI	Integer	Continuous	Ratio	CWT: Repolarization Peak mInimum
V2.RPI	Integer	Continuous	Ratio	CWT: Repolarization Peak mInimum
V3.RPI	Integer	Continuous	Ratio	CWT: Repolarization Peak mInimum
V4.RPI	Integer	Continuous	Ratio	CWT: Repolarization Peak mInimum
V5.RPI	Integer	Continuous	Ratio	CWT: Repolarization Peak mInimum
V6.RPI	Integer	Continuous	Ratio	CWT: Repolarization Peak mInimum
I.freq_RPI	Integer	Continuous	Freq. ID	CWT: Frequency ID at which peak RPI measure was found.
II.freq_RPI	Integer	Continuous	Freq. ID	CWT: Frequency ID at which peak RPI measure was found.
III.freq_RPI	Integer	Continuous	Freq. ID	CWT: Frequency ID at which peak RPI measure was found.
aVR.freq_RPI	Integer	Continuous	Freq. ID	CWT: Frequency ID at which peak RPI measure was found.
aVL.freq_RPI	Integer	Continuous	Freq. ID	CWT: Frequency ID at which peak RPI measure was found.
aVF.freq_RPI	Integer	Continuous	Freq. ID	CWT: Frequency ID at which peak RPI measure was found.
V1.freq_RPI	Integer	Continuous	Freq. ID	CWT: Frequency ID at which peak RPI measure was found.
V2.freq_RPI	Integer	Continuous	Freq. ID	CWT: Frequency ID at which peak RPI measure was found.
V3.freq_RPI	Integer	Continuous	Freq. ID	CWT: Frequency ID at which peak RPI measure was found.
V4.freq_RPI	Integer	Continuous	Freq. ID	CWT: Frequency ID at which peak RPI measure was found.
V5.freq_RPI	Integer	Continuous	Freq. ID	CWT: Frequency ID at which peak RPI measure was found.
V6.freq_RPI	Integer	Continuous	Freq. ID	CWT: Frequency ID at which peak RPI measure was found.
I.RPA	Integer	Continuous	Ratio	CWT: Repolarization Peak mAximum
II.RPA	Integer	Continuous	Ratio	CWT: Repolarization Peak mAximum
III.RPA	Integer	Continuous	Ratio	CWT: Repolarization Peak mAximum
aVR.RPA	Integer	Continuous	Ratio	CWT: Repolarization Peak mAximum
aVL.RPA	Integer	Continuous	Ratio	CWT: Repolarization Peak mAximum
aVF.RPA	Integer	Continuous	Ratio	CWT: Repolarization Peak mAximum
V1.RPA	Integer	Continuous	Ratio	CWT: Repolarization Peak mAximum
V2.RPA	Integer	Continuous	Ratio	CWT: Repolarization Peak mAximum
V3.RPA	Integer	Continuous	Ratio	CWT: Repolarization Peak mAximum
V4.RPA	Integer	Continuous	Ratio	CWT: Repolarization Peak mAximum
V5.RPA	Integer	Continuous	Ratio	CWT: Repolarization Peak mAximum
V6.RPA	Integer	Continuous	Ratio	CWT: Repolarization Peak mAximum
I.freq_RPA	Integer	Continuous	Freq. ID	CWT: Frequency ID at which peak RPA measure was found.
II.freq_RPA	Integer	Continuous	Freq. ID	CWT: Frequency ID at which peak RPA measure was found.
III.freq_RPA	Integer	Continuous	Freq. ID	CWT: Frequency ID at which peak RPA measure was found.
aVR.freq_RPA	Integer	Continuous	Freq. ID	CWT: Frequency ID at which peak RPA measure was found.
aVL.freq_RPA	Integer	Continuous	Freq. ID	CWT: Frequency ID at which peak RPA measure was found.
aVF.freq_RPA	Integer	Continuous	Freq. ID	CWT: Frequency ID at which peak RPA measure was found.
V1.freq_RPA	Integer	Continuous	Freq. ID	CWT: Frequency ID at which peak RPA measure was found.
V2.freq_RPA	Integer	Continuous	Freq. ID	CWT: Frequency ID at which peak RPA measure was found.
V3.freq_RPA	Integer	Continuous	Freq. ID	CWT: Frequency ID at which peak RPA measure was found.
V4.freq_RPA	Integer	Continuous	Freq. ID	CWT: Frequency ID at which peak RPA measure was found.
V5.freq_RPA	Integer	Continuous	Freq. ID	CWT: Frequency ID at which peak RPA measure was found.
V6.freq_RPA	Integer	Continuous	Freq. ID	CWT: Frequency ID at which peak RPA measure was found.
I.RLI	Integer	Continuous	Ratio	CWT: Repolarization Late mInimum
II.RLI	Integer	Continuous	Ratio	CWT: Repolarization Late mInimum
III.RLI	Integer	Continuous	Ratio	CWT: Repolarization Late mInimum
aVR.RLI	Integer	Continuous	Ratio	CWT: Repolarization Late mInimum
aVL.RLI	Integer	Continuous	Ratio	CWT: Repolarization Late mInimum

aVF.RLI	Integer	Continuous	Ratio	CWT: Repolarization Late mInimum
V1.RLI	Integer	Continuous	Ratio	CWT: Repolarization Late mInimum
V2.RLI	Integer	Continuous	Ratio	CWT: Repolarization Late mInimum
V3.RLI	Integer	Continuous	Ratio	CWT: Repolarization Late mInimum
V4.RLI	Integer	Continuous	Ratio	CWT: Repolarization Late mInimum
V5.RLI	Integer	Continuous	Ratio	CWT: Repolarization Late mInimum
V6.RLI	Integer	Continuous	Ratio	CWT: Repolarization Late mInimum
I.freq_RLI	Integer	Continuous	Freq. ID	CWT: Frequency ID at which peak RLI measure was found.
II.freq_RLI	Integer	Continuous	Freq. ID	CWT: Frequency ID at which peak RLI measure was found.
III.freq_RLI	Integer	Continuous	Freq. ID	CWT: Frequency ID at which peak RLI measure was found.
aVR.freq_RLI	Integer	Continuous	Freq. ID	CWT: Frequency ID at which peak RLI measure was found.
aVL.freq_RLI	Integer	Continuous	Freq. ID	CWT: Frequency ID at which peak RLI measure was found.
aVF.freq_RLI	Integer	Continuous	Freq. ID	CWT: Frequency ID at which peak RLI measure was found.
V1.freq_RLI	Integer	Continuous	Freq. ID	CWT: Frequency ID at which peak RLI measure was found.
V2.freq_RLI	Integer	Continuous	Freq. ID	CWT: Frequency ID at which peak RLI measure was found.
V3.freq_RLI	Integer	Continuous	Freq. ID	CWT: Frequency ID at which peak RLI measure was found.
V4.freq_RLI	Integer	Continuous	Freq. ID	CWT: Frequency ID at which peak RLI measure was found.
V5.freq_RLI	Integer	Continuous	Freq. ID	CWT: Frequency ID at which peak RLI measure was found.
V6.freq_RLI	Integer	Continuous	Freq. ID	CWT: Frequency ID at which peak RLI measure was found.
I.RLA	Integer	Continuous	Ratio	CWT: Repolarization Late mAximum
II.RLA	Integer	Continuous	Ratio	CWT: Repolarization Late mAximum
III.RLA	Integer	Continuous	Ratio	CWT: Repolarization Late mAximum
aVR.RLA	Integer	Continuous	Ratio	CWT: Repolarization Late mAximum
aVL.RLA	Integer	Continuous	Ratio	CWT: Repolarization Late mAximum
aVF.RLA	Integer	Continuous	Ratio	CWT: Repolarization Late mAximum
V1.RLA	Integer	Continuous	Ratio	CWT: Repolarization Late mAximum
V2.RLA	Integer	Continuous	Ratio	CWT: Repolarization Late mAximum
V3.RLA	Integer	Continuous	Ratio	CWT: Repolarization Late mAximum
V4.RLA	Integer	Continuous	Ratio	CWT: Repolarization Late mAximum
V5.RLA	Integer	Continuous	Ratio	CWT: Repolarization Late mAximum
V6.RLA	Integer	Continuous	Ratio	CWT: Repolarization Late mAximum
I.freq_RLA	Integer	Continuous	Freq. ID	CWT: Frequency ID at which peak RLA measure was found.
II.freq_RLA	Integer	Continuous	Freq. ID	CWT: Frequency ID at which peak RLA measure was found.
III.freq_RLA	Integer	Continuous	Freq. ID	CWT: Frequency ID at which peak RLA measure was found.
aVR.freq_RLA	Integer	Continuous	Freq. ID	CWT: Frequency ID at which peak RLA measure was found.
aVL.freq_RLA	Integer	Continuous	Freq. ID	CWT: Frequency ID at which peak RLA measure was found.
aVF.freq_RLA	Integer	Continuous	Freq. ID	CWT: Frequency ID at which peak RLA measure was found.
V1.freq_RLA	Integer	Continuous	Freq. ID	CWT: Frequency ID at which peak RLA measure was found.
V2.freq_RLA	Integer	Continuous	Freq. ID	CWT: Frequency ID at which peak RLA measure was found.
V3.freq_RLA	Integer	Continuous	Freq. ID	CWT: Frequency ID at which peak RLA measure was found.
V4.freq_RLA	Integer	Continuous	Freq. ID	CWT: Frequency ID at which peak RLA measure was found.
V5.freq_RLA	Integer	Continuous	Freq. ID	CWT: Frequency ID at which peak RLA measure was found.
V6.freq_RLA	Integer	Continuous	Freq. ID	CWT: Frequency ID at which peak RLA measure was found.
I.Rsign	Binary	Binary	N/A	CWT: Polarity of the “R-wave” (0=negative, 1=positive).
II.Rsign	Binary	Binary	N/A	CWT: Polarity of the “R-wave” (0=negative, 1=positive).
III.Rsign	Binary	Binary	N/A	CWT: Polarity of the “R-wave” (0=negative, 1=positive).
aVR.Rsign	Binary	Binary	N/A	CWT: Polarity of the “R-wave” (0=negative, 1=positive).
aVL.Rsign	Binary	Binary	N/A	CWT: Polarity of the “R-wave” (0=negative, 1=positive).
aVF.Rsign	Binary	Binary	N/A	CWT: Polarity of the “R-wave” (0=negative, 1=positive).

V1.Rsign	Binary	Binary	N/A	CWT: Polarity of the “R-wave” (0=negative, 1=positive).
V2.Rsign	Binary	Binary	N/A	CWT: Polarity of the “R-wave” (0=negative, 1=positive).
V3.Rsign	Binary	Binary	N/A	CWT: Polarity of the “R-wave” (0=negative, 1=positive).
V4.Rsign	Binary	Binary	N/A	CWT: Polarity of the “R-wave” (0=negative, 1=positive).
V5.Rsign	Binary	Binary	N/A	CWT: Polarity of the “R-wave” (0=negative, 1=positive).
V6.Rsign	Binary	Binary	N/A	CWT: Polarity of the “R-wave” (0=negative, 1=positive).
I.RAM	Floating Point	Continuous	N/A	CWT: RAM = (REM+RLM)/heartrate
II.RAM	Floating Point	Continuous	N/A	CWT: RAM = (REM+RLM)/heartrate
III.RAM	Floating Point	Continuous	N/A	CWT: RAM = (REM+RLM)/heartrate
aVR.RAM	Floating Point	Continuous	N/A	CWT: RAM = (REM+RLM)/heartrate
aVL.RAM	Floating Point	Continuous	N/A	CWT: RAM = (REM+RLM)/heartrate
aVF.RAM	Floating Point	Continuous	N/A	CWT: RAM = (REM+RLM)/heartrate
V1.RAM	Floating Point	Continuous	N/A	CWT: RAM = (REM+RLM)/heartrate
V2.RAM	Floating Point	Continuous	N/A	CWT: RAM = (REM+RLM)/heartrate
V3.RAM	Floating Point	Continuous	N/A	CWT: RAM = (REM+RLM)/heartrate
V4.RAM	Floating Point	Continuous	N/A	CWT: RAM = (REM+RLM)/heartrate
V5.RAM	Floating Point	Continuous	N/A	CWT: RAM = (REM+RLM)/heartrate
V6.RAM	Floating Point	Continuous	N/A	CWT: RAM = (REM+RLM)/heartrate
I.DAM	Floating Point	Continuous	N/A	CWT: DAM = (DOM+DPM+DTM)/heartrate
II.DAM	Floating Point	Continuous	N/A	CWT: DAM = (DOM+DPM+DTM)/heartrate
III.DAM	Floating Point	Continuous	N/A	CWT: DAM = (DOM+DPM+DTM)/heartrate
aVR.DAM	Floating Point	Continuous	N/A	CWT: DAM = (DOM+DPM+DTM)/heartrate
aVL.DAM	Floating Point	Continuous	N/A	CWT: DAM = (DOM+DPM+DTM)/heartrate
aVF.DAM	Floating Point	Continuous	N/A	CWT: DAM = (DOM+DPM+DTM)/heartrate
V1.DAM	Floating Point	Continuous	N/A	CWT: DAM = (DOM+DPM+DTM)/heartrate
V2.DAM	Floating Point	Continuous	N/A	CWT: DAM = (DOM+DPM+DTM)/heartrate
V3.DAM	Floating Point	Continuous	N/A	CWT: DAM = (DOM+DPM+DTM)/heartrate
V4.DAM	Floating Point	Continuous	N/A	CWT: DAM = (DOM+DPM+DTM)/heartrate
V5.DAM	Floating Point	Continuous	N/A	CWT: DAM = (DOM+DPM+DTM)/heartrate
V6.DAM	Floating Point	Continuous	N/A	CWT: DAM = (DOM+DPM+DTM)/heartrate
I.DRate	Floating Point	Continuous	Ratio	CWT: Depolarization Repolarization Rate, DRRate = RAM/DAM
II.DRate	Floating Point	Continuous	Ratio	CWT: Depolarization Repolarization Rate, DRRate = RAM/DAM
III.DRate	Floating Point	Continuous	Ratio	CWT: Depolarization Repolarization Rate, DRRate = RAM/DAM
aVR.DRate	Floating Point	Continuous	Ratio	CWT: Depolarization Repolarization Rate, DRRate = RAM/DAM
aVL.DRate	Floating Point	Continuous	Ratio	CWT: Depolarization Repolarization Rate, DRRate = RAM/DAM
aVF.DRate	Floating Point	Continuous	Ratio	CWT: Depolarization Repolarization Rate, DRRate = RAM/DAM
V1.DRate	Floating Point	Continuous	Ratio	CWT: Depolarization Repolarization Rate, DRRate = RAM/DAM
V2.DRate	Floating Point	Continuous	Ratio	CWT: Depolarization Repolarization Rate, DRRate = RAM/DAM
V3.DRate	Floating Point	Continuous	Ratio	CWT: Depolarization Repolarization Rate, DRRate = RAM/DAM
V4.DRate	Floating Point	Continuous	Ratio	CWT: Depolarization Repolarization Rate, DRRate = RAM/DAM
V5.DRate	Floating Point	Continuous	Ratio	CWT: Depolarization Repolarization Rate, DRRate = RAM/DAM
V6.DRate	Floating Point	Continuous	Ratio	CWT: Depolarization Repolarization Rate, DRRate = RAM/DAM
I.P21.sign	Binary (Char)	Binary	Sign	PowerSpectrum: Sign of Ratio of 2nd harmonic peak to the 1st harmonic peak.
II.P21.sign	Binary (Char)	Binary	Sign	PowerSpectrum: Sign of Ratio of 2nd harmonic peak to the 1st harmonic peak.
III.P21.sign	Binary (Char)	Binary	Sign	PowerSpectrum: Sign of Ratio of 2nd harmonic peak to the 1st harmonic peak.
aVR.P21.sign	Binary (Char)	Binary	Sign	PowerSpectrum: Sign of Ratio of 2nd harmonic peak to the 1st harmonic peak.
aVL.P21.sign	Binary (Char)	Binary	Sign	PowerSpectrum: Sign of Ratio of 2nd harmonic peak to the 1st harmonic peak.
aVF.P21.sign	Binary (Char)	Binary	Sign	PowerSpectrum: Sign of Ratio of 2nd harmonic peak to the 1st harmonic peak.
V1.P21.sign	Binary (Char)	Binary	Sign	PowerSpectrum: Sign of Ratio of 2nd harmonic peak to the 1st harmonic peak.

V2.P21.sign	Binary (Char)	Binary	Sign	PowerSpectrum: Sign of Ratio of 2nd harmonic peak to the 1st harmonic peak.
V3.P21.sign	Binary (Char)	Binary	Sign	PowerSpectrum: Sign of Ratio of 2nd harmonic peak to the 1st harmonic peak.
V4.P21.sign	Binary (Char)	Binary	Sign	PowerSpectrum: Sign of Ratio of 2nd harmonic peak to the 1st harmonic peak.
V5.P21.sign	Binary (Char)	Binary	Sign	PowerSpectrum: Sign of Ratio of 2nd harmonic peak to the 1st harmonic peak.
V6.P21.sign	Binary (Char)	Binary	Sign	PowerSpectrum: Sign of Ratio of 2nd harmonic peak to the 1st harmonic peak.
I.P51.sign	Binary (Char)	Binary	Sign	PowerSpectrum: Sign of Ratio of 5nd harmonic peak to the 1st harmonic peak.
II.P51.sign	Binary (Char)	Binary	Sign	PowerSpectrum: Sign of Ratio of 5nd harmonic peak to the 1st harmonic peak.
III.P51.sign	Binary (Char)	Binary	Sign	PowerSpectrum: Sign of Ratio of 5nd harmonic peak to the 1st harmonic peak.
aVR.P51.sign	Binary (Char)	Binary	Sign	PowerSpectrum: Sign of Ratio of 5nd harmonic peak to the 1st harmonic peak.
aVL.P51.sign	Binary (Char)	Binary	Sign	PowerSpectrum: Sign of Ratio of 5nd harmonic peak to the 1st harmonic peak.
aVF.P51.sign	Binary (Char)	Binary	Sign	PowerSpectrum: Sign of Ratio of 5nd harmonic peak to the 1st harmonic peak.
V1.P51.sign	Binary (Char)	Binary	Sign	PowerSpectrum: Sign of Ratio of 5nd harmonic peak to the 1st harmonic peak.
V2.P51.sign	Binary (Char)	Binary	Sign	PowerSpectrum: Sign of Ratio of 5nd harmonic peak to the 1st harmonic peak.
V3.P51.sign	Binary (Char)	Binary	Sign	PowerSpectrum: Sign of Ratio of 5nd harmonic peak to the 1st harmonic peak.
V4.P51.sign	Binary (Char)	Binary	Sign	PowerSpectrum: Sign of Ratio of 5nd harmonic peak to the 1st harmonic peak.
V5.P51.sign	Binary (Char)	Binary	Sign	PowerSpectrum: Sign of Ratio of 5nd harmonic peak to the 1st harmonic peak.
V6.P51.sign	Binary (Char)	Binary	Sign	PowerSpectrum: Sign of Ratio of 5nd harmonic peak to the 1st harmonic peak.
I.P43.sign	Binary (Char)	Binary	Sign	PowerSpectrum: Sign of Ratio of 4th harmonic peak to the 3rd harmonic peak.
II.P43.sign	Binary (Char)	Binary	Sign	PowerSpectrum: Sign of Ratio of 4th harmonic peak to the 3rd harmonic peak.
III.P43.sign	Binary (Char)	Binary	Sign	PowerSpectrum: Sign of Ratio of 4th harmonic peak to the 3rd harmonic peak.
aVR.P43.sign	Binary (Char)	Binary	Sign	PowerSpectrum: Sign of Ratio of 4th harmonic peak to the 3rd harmonic peak.
aVL.P43.sign	Binary (Char)	Binary	Sign	PowerSpectrum: Sign of Ratio of 4th harmonic peak to the 3rd harmonic peak.
aVF.P43.sign	Binary (Char)	Binary	Sign	PowerSpectrum: Sign of Ratio of 4th harmonic peak to the 3rd harmonic peak.
V1.P43.sign	Binary (Char)	Binary	Sign	PowerSpectrum: Sign of Ratio of 4th harmonic peak to the 3rd harmonic peak.
V2.P43.sign	Binary (Char)	Binary	Sign	PowerSpectrum: Sign of Ratio of 4th harmonic peak to the 3rd harmonic peak.
V3.P43.sign	Binary (Char)	Binary	Sign	PowerSpectrum: Sign of Ratio of 4th harmonic peak to the 3rd harmonic peak.
V4.P43.sign	Binary (Char)	Binary	Sign	PowerSpectrum: Sign of Ratio of 4th harmonic peak to the 3rd harmonic peak.
V5.P43.sign	Binary (Char)	Binary	Sign	PowerSpectrum: Sign of Ratio of 4th harmonic peak to the 3rd harmonic peak.
V6.P43.sign	Binary (Char)	Binary	Sign	PowerSpectrum: Sign of Ratio of 4th harmonic peak to the 3rd harmonic peak.
I.P53.sign	Binary (Char)	Binary	Sign	PowerSpectrum: Sign of Ratio of 5th harmonic peak to the 3rd harmonic peak.
II.P53.sign	Binary (Char)	Binary	Sign	PowerSpectrum: Sign of Ratio of 5th harmonic peak to the 3rd harmonic peak.
III.P53.sign	Binary (Char)	Binary	Sign	PowerSpectrum: Sign of Ratio of 5th harmonic peak to the 3rd harmonic peak.
aVR.P53.sign	Binary (Char)	Binary	Sign	PowerSpectrum: Sign of Ratio of 5th harmonic peak to the 3rd harmonic peak.
aVL.P53.sign	Binary (Char)	Binary	Sign	PowerSpectrum: Sign of Ratio of 5th harmonic peak to the 3rd harmonic peak.
aVF.P53.sign	Binary (Char)	Binary	Sign	PowerSpectrum: Sign of Ratio of 5th harmonic peak to the 3rd harmonic peak.
V1.P53.sign	Binary (Char)	Binary	Sign	PowerSpectrum: Sign of Ratio of 5th harmonic peak to the 3rd harmonic peak.
V2.P53.sign	Binary (Char)	Binary	Sign	PowerSpectrum: Sign of Ratio of 5th harmonic peak to the 3rd harmonic peak.
V3.P53.sign	Binary (Char)	Binary	Sign	PowerSpectrum: Sign of Ratio of 5th harmonic peak to the 3rd harmonic peak.
V4.P53.sign	Binary (Char)	Binary	Sign	PowerSpectrum: Sign of Ratio of 5th harmonic peak to the 3rd harmonic peak.
V5.P53.sign	Binary (Char)	Binary	Sign	PowerSpectrum: Sign of Ratio of 5th harmonic peak to the 3rd harmonic peak.
V6.P53.sign	Binary (Char)	Binary	Sign	PowerSpectrum: Sign of Ratio of 5th harmonic peak to the 3rd harmonic peak.
I.LO1.sign	Binary (Char)	Binary	Sign	PowerSpectrum: Sign of Ratio of 1st harmonic energy to total energy.
II.LO1.sign	Binary (Char)	Binary	Sign	PowerSpectrum: Sign of Ratio of 1st harmonic energy to total energy.
III.LO1.sign	Binary (Char)	Binary	Sign	PowerSpectrum: Sign of Ratio of 1st harmonic energy to total energy.
aVR.LO1.sign	Binary (Char)	Binary	Sign	PowerSpectrum: Sign of Ratio of 1st harmonic energy to total energy.
aVL.LO1.sign	Binary (Char)	Binary	Sign	PowerSpectrum: Sign of Ratio of 1st harmonic energy to total energy.
aVF.LO1.sign	Binary (Char)	Binary	Sign	PowerSpectrum: Sign of Ratio of 1st harmonic energy to total energy.
V1.LO1.sign	Binary (Char)	Binary	Sign	PowerSpectrum: Sign of Ratio of 1st harmonic energy to total energy.
V2.LO1.sign	Binary (Char)	Binary	Sign	PowerSpectrum: Sign of Ratio of 1st harmonic energy to total energy.

V3.LO1.sign	Binary (Char)	Binary	Sign	PowerSpectrum: Sign of Ratio of 1st harmonic energy to total energy.
V4.LO1.sign	Binary (Char)	Binary	Sign	PowerSpectrum: Sign of Ratio of 1st harmonic energy to total energy.
V5.LO1.sign	Binary (Char)	Binary	Sign	PowerSpectrum: Sign of Ratio of 1st harmonic energy to total energy.
V6.LO1.sign	Binary (Char)	Binary	Sign	PowerSpectrum: Sign of Ratio of 1st harmonic energy to total energy.
I.LO3.sign	Binary (Char)	Binary	Sign	PowerSpectrum: Sign of Ratio of 3rd harmonic energy to total energy.
II.LO3.sign	Binary (Char)	Binary	Sign	PowerSpectrum: Sign of Ratio of 3rd harmonic energy to total energy.
III.LO3.sign	Binary (Char)	Binary	Sign	PowerSpectrum: Sign of Ratio of 3rd harmonic energy to total energy.
aVR.LO3.sign	Binary (Char)	Binary	Sign	PowerSpectrum: Sign of Ratio of 3rd harmonic energy to total energy.
aVL.LO3.sign	Binary (Char)	Binary	Sign	PowerSpectrum: Sign of Ratio of 3rd harmonic energy to total energy.
aVF.LO3.sign	Binary (Char)	Binary	Sign	PowerSpectrum: Sign of Ratio of 3rd harmonic energy to total energy.
V1.LO3.sign	Binary (Char)	Binary	Sign	PowerSpectrum: Sign of Ratio of 3rd harmonic energy to total energy.
V2.LO3.sign	Binary (Char)	Binary	Sign	PowerSpectrum: Sign of Ratio of 3rd harmonic energy to total energy.
V3.LO3.sign	Binary (Char)	Binary	Sign	PowerSpectrum: Sign of Ratio of 3rd harmonic energy to total energy.
V4.LO3.sign	Binary (Char)	Binary	Sign	PowerSpectrum: Sign of Ratio of 3rd harmonic energy to total energy.
V5.LO3.sign	Binary (Char)	Binary	Sign	PowerSpectrum: Sign of Ratio of 3rd harmonic energy to total energy.
V6.LO3.sign	Binary (Char)	Binary	Sign	PowerSpectrum: Sign of Ratio of 3rd harmonic energy to total energy.
I.LOA.sign	Binary (Char)	Binary	Sign	PowerSpectrum: Sign of Ratio of 1-4 harmonic energy to total energy.
II.LOA.sign	Binary (Char)	Binary	Sign	PowerSpectrum: Sign of Ratio of 1-4 harmonic energy to total energy.
III.LOA.sign	Binary (Char)	Binary	Sign	PowerSpectrum: Sign of Ratio of 1-4 harmonic energy to total energy.
aVR.LOA.sign	Binary (Char)	Binary	Sign	PowerSpectrum: Sign of Ratio of 1-4 harmonic energy to total energy.
aVL.LOA.sign	Binary (Char)	Binary	Sign	PowerSpectrum: Sign of Ratio of 1-4 harmonic energy to total energy.
aVF.LOA.sign	Binary (Char)	Binary	Sign	PowerSpectrum: Sign of Ratio of 1-4 harmonic energy to total energy.
V1.LOA.sign	Binary (Char)	Binary	Sign	PowerSpectrum: Sign of Ratio of 1-4 harmonic energy to total energy.
V2.LOA.sign	Binary (Char)	Binary	Sign	PowerSpectrum: Sign of Ratio of 1-4 harmonic energy to total energy.
V3.LOA.sign	Binary (Char)	Binary	Sign	PowerSpectrum: Sign of Ratio of 1-4 harmonic energy to total energy.
V4.LOA.sign	Binary (Char)	Binary	Sign	PowerSpectrum: Sign of Ratio of 1-4 harmonic energy to total energy.
V5.LOA.sign	Binary (Char)	Binary	Sign	PowerSpectrum: Sign of Ratio of 1-4 harmonic energy to total energy.
V6.LOA.sign	Binary (Char)	Binary	Sign	PowerSpectrum: Sign of Ratio of 1-4 harmonic energy to total energy.
I.P21.value	Floating Point	Continuous	Ratio	PowerSpectrum: Ratio of 2nd harmonic peak to the 1st harmonic peak.
II.P21.value	Floating Point	Continuous	Ratio	PowerSpectrum: Ratio of 2nd harmonic peak to the 1st harmonic peak.
III.P21.value	Floating Point	Continuous	Ratio	PowerSpectrum: Ratio of 2nd harmonic peak to the 1st harmonic peak.
aVR.P21.value	Floating Point	Continuous	Ratio	PowerSpectrum: Ratio of 2nd harmonic peak to the 1st harmonic peak.
aVL.P21.value	Floating Point	Continuous	Ratio	PowerSpectrum: Ratio of 2nd harmonic peak to the 1st harmonic peak.
aVF.P21.value	Floating Point	Continuous	Ratio	PowerSpectrum: Ratio of 2nd harmonic peak to the 1st harmonic peak.
V1.P21.value	Floating Point	Continuous	Ratio	PowerSpectrum: Ratio of 2nd harmonic peak to the 1st harmonic peak.
V2.P21.value	Floating Point	Continuous	Ratio	PowerSpectrum: Ratio of 2nd harmonic peak to the 1st harmonic peak.
V3.P21.value	Floating Point	Continuous	Ratio	PowerSpectrum: Ratio of 2nd harmonic peak to the 1st harmonic peak.
V4.P21.value	Floating Point	Continuous	Ratio	PowerSpectrum: Ratio of 2nd harmonic peak to the 1st harmonic peak.
V5.P21.value	Floating Point	Continuous	Ratio	PowerSpectrum: Ratio of 2nd harmonic peak to the 1st harmonic peak.
V6.P21.value	Floating Point	Continuous	Ratio	PowerSpectrum: Ratio of 2nd harmonic peak to the 1st harmonic peak.
I.P51.value	Floating Point	Continuous	Ratio	PowerSpectrum: Ratio of 5th harmonic peak to the 1st harmonic peak.
II.P51.value	Floating Point	Continuous	Ratio	PowerSpectrum: Ratio of 5th harmonic peak to the 1st harmonic peak.
III.P51.value	Floating Point	Continuous	Ratio	PowerSpectrum: Ratio of 5th harmonic peak to the 1st harmonic peak.
aVR.P51.value	Floating Point	Continuous	Ratio	PowerSpectrum: Ratio of 5th harmonic peak to the 1st harmonic peak.
aVL.P51.value	Floating Point	Continuous	Ratio	PowerSpectrum: Ratio of 5th harmonic peak to the 1st harmonic peak.
aVF.P51.value	Floating Point	Continuous	Ratio	PowerSpectrum: Ratio of 5th harmonic peak to the 1st harmonic peak.
V1.P51.value	Floating Point	Continuous	Ratio	PowerSpectrum: Ratio of 5th harmonic peak to the 1st harmonic peak.
V2.P51.value	Floating Point	Continuous	Ratio	PowerSpectrum: Ratio of 5th harmonic peak to the 1st harmonic peak.
V3.P51.value	Floating Point	Continuous	Ratio	PowerSpectrum: Ratio of 5th harmonic peak to the 1st harmonic peak.

V4.P51.value	Floating Point	Continuous	Ratio	PowerSpectrum: Ratio of 5th harmonic peak to the 1st harmonic peak.
V5.P51.value	Floating Point	Continuous	Ratio	PowerSpectrum: Ratio of 5th harmonic peak to the 1st harmonic peak.
V6.P51.value	Floating Point	Continuous	Ratio	PowerSpectrum: Ratio of 5th harmonic peak to the 1st harmonic peak.
I.P43.value	Floating Point	Continuous	Ratio	PowerSpectrum: Ratio of 4th harmonic peak to the 3rd harmonic peak.
II.P43.value	Floating Point	Continuous	Ratio	PowerSpectrum: Ratio of 4th harmonic peak to the 3rd harmonic peak.
III.P43.value	Floating Point	Continuous	Ratio	PowerSpectrum: Ratio of 4th harmonic peak to the 3rd harmonic peak.
aVR.P43.value	Floating Point	Continuous	Ratio	PowerSpectrum: Ratio of 4th harmonic peak to the 3rd harmonic peak.
aVL.P43.value	Floating Point	Continuous	Ratio	PowerSpectrum: Ratio of 4th harmonic peak to the 3rd harmonic peak.
aVF.P43.value	Floating Point	Continuous	Ratio	PowerSpectrum: Ratio of 4th harmonic peak to the 3rd harmonic peak.
V1.P43.value	Floating Point	Continuous	Ratio	PowerSpectrum: Ratio of 4th harmonic peak to the 3rd harmonic peak.
V2.P43.value	Floating Point	Continuous	Ratio	PowerSpectrum: Ratio of 4th harmonic peak to the 3rd harmonic peak.
V3.P43.value	Floating Point	Continuous	Ratio	PowerSpectrum: Ratio of 4th harmonic peak to the 3rd harmonic peak.
V4.P43.value	Floating Point	Continuous	Ratio	PowerSpectrum: Ratio of 4th harmonic peak to the 3rd harmonic peak.
V5.P43.value	Floating Point	Continuous	Ratio	PowerSpectrum: Ratio of 4th harmonic peak to the 3rd harmonic peak.
V6.P43.value	Floating Point	Continuous	Ratio	PowerSpectrum: Ratio of 4th harmonic peak to the 3rd harmonic peak.
I.P53.value	Floating Point	Continuous	Ratio	PowerSpectrum: Ratio of 5th harmonic peak to the 3rd harmonic peak.
II.P53.value	Floating Point	Continuous	Ratio	PowerSpectrum: Ratio of 5th harmonic peak to the 3rd harmonic peak.
III.P53.value	Floating Point	Continuous	Ratio	PowerSpectrum: Ratio of 5th harmonic peak to the 3rd harmonic peak.
aVR.P53.value	Floating Point	Continuous	Ratio	PowerSpectrum: Ratio of 5th harmonic peak to the 3rd harmonic peak.
aVL.P53.value	Floating Point	Continuous	Ratio	PowerSpectrum: Ratio of 5th harmonic peak to the 3rd harmonic peak.
aVF.P53.value	Floating Point	Continuous	Ratio	PowerSpectrum: Ratio of 5th harmonic peak to the 3rd harmonic peak.
V1.P53.value	Floating Point	Continuous	Ratio	PowerSpectrum: Ratio of 5th harmonic peak to the 3rd harmonic peak.
V2.P53.value	Floating Point	Continuous	Ratio	PowerSpectrum: Ratio of 5th harmonic peak to the 3rd harmonic peak.
V3.P53.value	Floating Point	Continuous	Ratio	PowerSpectrum: Ratio of 5th harmonic peak to the 3rd harmonic peak.
V4.P53.value	Floating Point	Continuous	Ratio	PowerSpectrum: Ratio of 5th harmonic peak to the 3rd harmonic peak.
V5.P53.value	Floating Point	Continuous	Ratio	PowerSpectrum: Ratio of 5th harmonic peak to the 3rd harmonic peak.
V6.P53.value	Floating Point	Continuous	Ratio	PowerSpectrum: Ratio of 5th harmonic peak to the 3rd harmonic peak.
I.LO1.value	Floating Point	Continuous	Ratio	PowerSpectrum: Ratio of 1st harmonic energy to total energy.
II.LO1.value	Floating Point	Continuous	Ratio	PowerSpectrum: Ratio of 1st harmonic energy to total energy.
III.LO1.value	Floating Point	Continuous	Ratio	PowerSpectrum: Ratio of 1st harmonic energy to total energy.
aVR.LO1.value	Floating Point	Continuous	Ratio	PowerSpectrum: Ratio of 1st harmonic energy to total energy.
aVL.LO1.value	Floating Point	Continuous	Ratio	PowerSpectrum: Ratio of 1st harmonic energy to total energy.
aVF.LO1.value	Floating Point	Continuous	Ratio	PowerSpectrum: Ratio of 1st harmonic energy to total energy.
V1.LO1.value	Floating Point	Continuous	Ratio	PowerSpectrum: Ratio of 1st harmonic energy to total energy.
V2.LO1.value	Floating Point	Continuous	Ratio	PowerSpectrum: Ratio of 1st harmonic energy to total energy.
V3.LO1.value	Floating Point	Continuous	Ratio	PowerSpectrum: Ratio of 1st harmonic energy to total energy.
V4.LO1.value	Floating Point	Continuous	Ratio	PowerSpectrum: Ratio of 1st harmonic energy to total energy.
V5.LO1.value	Floating Point	Continuous	Ratio	PowerSpectrum: Ratio of 1st harmonic energy to total energy.
V6.LO1.value	Floating Point	Continuous	Ratio	PowerSpectrum: Ratio of 1st harmonic energy to total energy.
I.LO3.value	Floating Point	Continuous	Ratio	PowerSpectrum: Ratio of 3rd harmonic energy to total energy.
II.LO3.value	Floating Point	Continuous	Ratio	PowerSpectrum: Ratio of 3rd harmonic energy to total energy.
III.LO3.value	Floating Point	Continuous	Ratio	PowerSpectrum: Ratio of 3rd harmonic energy to total energy.
aVR.LO3.value	Floating Point	Continuous	Ratio	PowerSpectrum: Ratio of 3rd harmonic energy to total energy.
aVL.LO3.value	Floating Point	Continuous	Ratio	PowerSpectrum: Ratio of 3rd harmonic energy to total energy.
aVF.LO3.value	Floating Point	Continuous	Ratio	PowerSpectrum: Ratio of 3rd harmonic energy to total energy.
V1.LO3.value	Floating Point	Continuous	Ratio	PowerSpectrum: Ratio of 3rd harmonic energy to total energy.
V2.LO3.value	Floating Point	Continuous	Ratio	PowerSpectrum: Ratio of 3rd harmonic energy to total energy.
V3.LO3.value	Floating Point	Continuous	Ratio	PowerSpectrum: Ratio of 3rd harmonic energy to total energy.
V4.LO3.value	Floating Point	Continuous	Ratio	PowerSpectrum: Ratio of 3rd harmonic energy to total energy.

V5.LO3.value	Floating Point	Continuous	Ratio	PowerSpectrum: Ratio of 3rd harmonic energy to total energy.
V6.LO3.value	Floating Point	Continuous	Ratio	PowerSpectrum: Ratio of 3rd harmonic energy to total energy.
I.LOA.value	Floating Point	Continuous	Ratio	PowerSpectrum: Ratio of 1-4 harmonic energy to total energy.
II.LOA.value	Floating Point	Continuous	Ratio	PowerSpectrum: Ratio of 1-4 harmonic energy to total energy.
III.LOA.value	Floating Point	Continuous	Ratio	PowerSpectrum: Ratio of 1-4 harmonic energy to total energy.
aVR.LOA.value	Floating Point	Continuous	Ratio	PowerSpectrum: Ratio of 1-4 harmonic energy to total energy.
aVL.LOA.value	Floating Point	Continuous	Ratio	PowerSpectrum: Ratio of 1-4 harmonic energy to total energy.
aVF.LOA.value	Floating Point	Continuous	Ratio	PowerSpectrum: Ratio of 1-4 harmonic energy to total energy.
V1.LOA.value	Floating Point	Continuous	Ratio	PowerSpectrum: Ratio of 1-4 harmonic energy to total energy.
V2.LOA.value	Floating Point	Continuous	Ratio	PowerSpectrum: Ratio of 1-4 harmonic energy to total energy.
V3.LOA.value	Floating Point	Continuous	Ratio	PowerSpectrum: Ratio of 1-4 harmonic energy to total energy.
V4.LOA.value	Floating Point	Continuous	Ratio	PowerSpectrum: Ratio of 1-4 harmonic energy to total energy.
V5.LOA.value	Floating Point	Continuous	Ratio	PowerSpectrum: Ratio of 1-4 harmonic energy to total energy.
V6.LOA.value	Floating Point	Continuous	Ratio	PowerSpectrum: Ratio of 1-4 harmonic energy to total energy.
Glasgow Heart Rate	Integer	Continuous	bpm	Heart Rate taken from ECG data (in beats-per-minute)
RR Interval	Integer	Continuous	ms	Timing between beginning of QRS Complexes of two adjacent cardiac cycles. Inverse of Heart Rate (i.e. 1/HR).
PR Interval	Integer	Continuous	ms	Timing between P-onset and QRS onset.
QRS Duration	Integer	Continuous	ms	Timing between QRS onset and QRS termination.
QT Interval	Integer	Continuous	ms	Timing between QRS onset and T-wave end.
QTc Interval	Integer	Continuous	ms	Timing between QRS onset and T-wave end (corrected using Hodge's formula).
P Axis	Integer	Continuous	degrees	Major direction of the overall electrical activity of the P-Wave (the frontal plane axes calculated from leads I and III using the algebraic sum of the amplitudes - all axes are calculated using sum of deflections in leads I and III)
QRS Axis	Integer	Continuous	degrees	Major direction of the overall electrical activity of the QRS Complex (the frontal plane axes calculated from leads I and III using the algebraic sum of the amplitudes - all axes are calculated using sum of deflections in leads I and III)
T Axis	Integer	Continuous	degrees	Major direction of the overall electrical activity of the T-Wave (the frontal plane axes calculated from leads I and III using the algebraic sum of the amplitudes - all axes are calculated using sum of deflections in leads I and III)
QT Dispersion	Integer	Continuous	msec	QT Dispersion (as calculated by Glasgow).
Summary Group	Integer	Categorical	N/A	Glasgow statemend group number (1-18 = text group number; 0 = block separation)
Summary Type	Integer	Categorical	N/A	Glasgow statemend type (0 = reason; 1 = statement; 2 = additional statement)
Summary Code	Integer	Categorical	N/A	Glasgow code of the reason, statement or the additional statement
QrsFrontalAxis	Integer	Continuous	degrees	QRS axis (frontal) INTEGER [-180,+180]
STFrontalAxis	Integer	Continuous	degrees	ST frontal axis, INT: -180 to +180 [degrees]
Display Heart Rate	Integer	Continuous	bpm	
Sinus Rate	Integer	Continuous	bpm	
Ventricular Rate	Integer	Continuous	bpm	
Heart Rate Variability	Integer	Continuous	msec	
PFrontalAxis	Integer	Continuous	degrees	P frontal axis, INT: -180 to +180 [degrees]
TFrontalAxis	Integer	Continuous	degrees	T frontal axis, INT: -180 to +180 [degrees]
Sinus Average RR	Integer	Continuous	msec	Average RR interval
Ventricular Average RR	Integer	Continuous	msec	
StdDev Normal RR Intervals	Integer	Continuous	msec	
Qrs Pseudo Vector 4/8 Spatial Velocity	Integer	Continuous	uvolt/msec	
Qrs Pseudo Vector 5/8 Spatial Velocity	Integer	Continuous	uvolt/msec	
Qrs Pseudo Vector 6/8 Spatial Velocity	Integer	Continuous	uvolt/msec	
Qrs Pseudo Vector 7/8 Spatial Velocity	Integer	Continuous	uvolt/msec	

Qrs Pseudo Vector Maximum Amplitude	Integer	Continuous	uvolt/msec	Max QRS spatial velocity (12 lead)
LVH Score	Integer	Continuous	N/A	
LV Strain	Binary	Binary	N/A	
ST Duration	Integer	Continuous	msec	Overall ST duration
PR Interval	Integer	Continuous	msec	Overall PR interval
QT Interval	Integer	Continuous	msec	Overall QT interval
QT Dispersion	Integer	Continuous	msec	
QTc (used)	Integer	Continuous	msec	QTc (used by program)
QTc Formula (used)	String	Categorical	N/A	Hodge, Bazett, Fridericia, Framingham
P Terminal (V1)	Integer	Continuous	uvolt/msec	P terminal force in V1
QTc Hodge	Integer	Continuous	msec	
QTc Bazett	Integer	Continuous	msec	
QTc Fridericia	Integer	Continuous	msec	
QTc Framingham	Integer	Continuous	msec	
OverallPonset	Integer	Continuous	msec	
OverallPtermination	Integer	Continuous	msec	
OverallPduration	Integer	Continuous	msec	
OverallQRSonset	Integer	Continuous	msec	
OverallQRStermination	Integer	Continuous	msec	
OverallQRSduration	Integer	Continuous	msec	
OverallTonset	Integer	Continuous	msec	
OverallTtermination	Integer	Continuous	msec	
OverallTduration	Integer	Continuous	msec	
P waves found	String	Binary	Y/N	Yes, No
Indeterminate QRS axis	String	Binary	Y/N	Yes, No
Default gender used	String	Binary	Y/N	Yes, No
Default race used	String	Binary	Y/N	Yes, No
Default age used	String	Binary	Y/N	Yes, No
I. P Ons	Integer	Continuous	msec	
I. P Dur	Integer	Continuous	msec	
I. QRS Ons	Integer	Continuous	msec	
I. QRS Dur	Integer	Continuous	msec	
I. ST80 Amp	Integer	Continuous	microvolts	
I. Q Dur	Integer	Continuous	msec	
I. R Dur	Integer	Continuous	msec	
I. S Dur	Integer	Continuous	msec	
I. R' Dur	Integer	Continuous	msec	
I. S' Dur	Integer	Continuous	msec	
I. R" Dur	Integer	Continuous	msec	
I. S" Dur	Integer	Continuous	msec	
I. ST Dur	Integer	Continuous	msec	
I. T Ons	Integer	Continuous	msec	
I. T Dur	Integer	Continuous	msec	
I. P+ Dur	Integer	Continuous	msec	
I. T+ Dur	Integer	Continuous	msec	
I. QRS IntD	Integer	Continuous	msec	
I. P+ Amp	Integer	Continuous	microvolts	
I. P- Amp	Integer	Continuous	microvolts	
I. P2P Amp	Integer	Continuous	microvolts	

I. R1 Amp	Integer	Continuous	microvolts	
I. Q Amp	Integer	Continuous	microvolts	
I. R Amp	Integer	Continuous	microvolts	
I. S Amp	Integer	Continuous	microvolts	
I. R' Amp	Integer	Continuous	microvolts	
I. S' Amp	Integer	Continuous	microvolts	
I. R" Amp	Integer	Continuous	microvolts	
I. S" Amp	Integer	Continuous	microvolts	
I. ST Amp	Integer	Continuous	microvolts	
I. STT28 Amp	Integer	Continuous	microvolts	
I. STT38 Amp	Integer	Continuous	microvolts	
I. T+ Amp	Integer	Continuous	microvolts	
I. T- Amp	Integer	Continuous	microvolts	
I. QRS Area	Integer	Continuous	uvolt-msec/20	
I. P Area	Integer	Continuous	uvolt-msec/20	
I. T Area	Integer	Continuous	uvolt-msec/20	
I. P Morph	Integer	Categorical	N/A	P morphology (INT: -2 to +2)
I. T Morph	Integer	Categorical	N/A	T morphology (INT: -2 to +2)
I. R Notch	Integer	Continuous	N/A	R wave notches
I. DeltaConf	Integer	Continuous	percent	Delta wave confidence (INT: 0 to 100)
I. ST Slope	Integer	Continuous	degrees	ST slope (INT: - 90 to + 90)
I. QT Int	Integer	Continuous	msec	QT interval
II. P Ons	Integer	Continuous	msec	
II. P Dur	Integer	Continuous	msec	
II. QRS Ons	Integer	Continuous	msec	
II. QRS Dur	Integer	Continuous	msec	
II. ST80 Amp	Integer	Continuous	microvolts	
II. Q Dur	Integer	Continuous	msec	
II. R Dur	Integer	Continuous	msec	
II. S Dur	Integer	Continuous	msec	
II. R' Dur	Integer	Continuous	msec	
II. S' Dur	Integer	Continuous	msec	
II. R" Dur	Integer	Continuous	msec	
II. S" Dur	Integer	Continuous	msec	
II. ST Dur	Integer	Continuous	msec	
II. T Ons	Integer	Continuous	msec	
II. T Dur	Integer	Continuous	msec	
II. P+ Dur	Integer	Continuous	msec	
II. T+ Dur	Integer	Continuous	msec	
II. QRS IntD	Integer	Continuous	msec	
II. P+ Amp	Integer	Continuous	microvolts	
II. P- Amp	Integer	Continuous	microvolts	
II. P2P Amp	Integer	Continuous	microvolts	
II. R1 Amp	Integer	Continuous	microvolts	
II. Q Amp	Integer	Continuous	microvolts	
II. R Amp	Integer	Continuous	microvolts	
II. S Amp	Integer	Continuous	microvolts	
II. R' Amp	Integer	Continuous	microvolts	
II. S' Amp	Integer	Continuous	microvolts	

II. R" Amp	Integer	Continuous	microvolts	
II. S" Amp	Integer	Continuous	microvolts	
II. ST Amp	Integer	Continuous	microvolts	
II. STT28 Amp	Integer	Continuous	microvolts	
II. STT38 Amp	Integer	Continuous	microvolts	
II. T+ Amp	Integer	Continuous	microvolts	
II. T- Amp	Integer	Continuous	microvolts	
II. QRS Area	Integer	Continuous	uvolt-msec/20	
II. P Area	Integer	Continuous	uvolt-msec/20	
II. T Area	Integer	Continuous	uvolt-msec/20	
II. P Morph	Integer	Categorical	N/A	P morphology (INT: -2 to +2)
II. T Morph	Integer	Categorical	N/A	T morphology (INT: -2 to +2)
II. R Notch	Integer	Continuous	N/A	R wave notches
II. DeltaConf	Integer	Continuous	percent	Delta wave confidence (INT: 0 to 100)
II. ST Slope	Integer	Continuous	degrees	ST slope (INT: - 90 to + 90)
II. QT Int	Integer	Continuous	msec	QT interval
III. P Ons	Integer	Continuous	msec	
III. P Dur	Integer	Continuous	msec	
III. QRS Ons	Integer	Continuous	msec	
III. QRS Dur	Integer	Continuous	msec	
III. ST80 Amp	Integer	Continuous	microvolts	
III. Q Dur	Integer	Continuous	msec	
III. R Dur	Integer	Continuous	msec	
III. S Dur	Integer	Continuous	msec	
III. R' Dur	Integer	Continuous	msec	
III. S' Dur	Integer	Continuous	msec	
III. R" Dur	Integer	Continuous	msec	
III. S" Dur	Integer	Continuous	msec	
III. ST Dur	Integer	Continuous	msec	
III. T Ons	Integer	Continuous	msec	
III. T Dur	Integer	Continuous	msec	
III. P+ Dur	Integer	Continuous	msec	
III. T+ Dur	Integer	Continuous	msec	
III. QRS IntD	Integer	Continuous	msec	
III. P+ Amp	Integer	Continuous	microvolts	
III. P- Amp	Integer	Continuous	microvolts	
III. P2P Amp	Integer	Continuous	microvolts	
III. R1 Amp	Integer	Continuous	microvolts	
III. Q Amp	Integer	Continuous	microvolts	
III. R Amp	Integer	Continuous	microvolts	
III. S Amp	Integer	Continuous	microvolts	
III. R' Amp	Integer	Continuous	microvolts	
III. S' Amp	Integer	Continuous	microvolts	
III. R" Amp	Integer	Continuous	microvolts	
III. S" Amp	Integer	Continuous	microvolts	
III. ST Amp	Integer	Continuous	microvolts	
III. STT28 Amp	Integer	Continuous	microvolts	
III. STT38 Amp	Integer	Continuous	microvolts	
III. T+ Amp	Integer	Continuous	microvolts	

III. T- Amp	Integer	Continuous	microvolts	
III. QRS Area	Integer	Continuous	uvolt-msec/20	
III. P Area	Integer	Continuous	uvolt-msec/20	
III. T Area	Integer	Continuous	uvolt-msec/20	
III. P Morph	Integer	Categorical	N/A	P morphology (INT: -2 to +2)
III. T Morph	Integer	Categorical	N/A	T morphology (INT: -2 to +2)
III. R Notch	Integer	Continuous	N/A	R wave notches
III. DeltaConf	Integer	Continuous	percent	Delta wave confidence (INT: 0 to 100)
III. ST Slope	Integer	Continuous	degrees	ST slope (INT: - 90 to + 90)
III. QT Int	Integer	Continuous	msec	QT interval
aVR. P Ons	Integer	Continuous	msec	
aVR. P Dur	Integer	Continuous	msec	
aVR. QRS Ons	Integer	Continuous	msec	
aVR. QRS Dur	Integer	Continuous	msec	
aVR. ST80 Amp	Integer	Continuous	microvolts	
aVR. Q Dur	Integer	Continuous	msec	
aVR. R Dur	Integer	Continuous	msec	
aVR. S Dur	Integer	Continuous	msec	
aVR. R' Dur	Integer	Continuous	msec	
aVR. S' Dur	Integer	Continuous	msec	
aVR. R" Dur	Integer	Continuous	msec	
aVR. S" Dur	Integer	Continuous	msec	
aVR. ST Dur	Integer	Continuous	msec	
aVR. T Ons	Integer	Continuous	msec	
aVR. T Dur	Integer	Continuous	msec	
aVR. P+ Dur	Integer	Continuous	msec	
aVR. T+ Dur	Integer	Continuous	msec	
aVR. QRS IntD	Integer	Continuous	msec	
aVR. P+ Amp	Integer	Continuous	microvolts	
aVR. P- Amp	Integer	Continuous	microvolts	
aVR. P2P Amp	Integer	Continuous	microvolts	
aVR. R1 Amp	Integer	Continuous	microvolts	
aVR. Q Amp	Integer	Continuous	microvolts	
aVR. R Amp	Integer	Continuous	microvolts	
aVR. S Amp	Integer	Continuous	microvolts	
aVR. R' Amp	Integer	Continuous	microvolts	
aVR. S' Amp	Integer	Continuous	microvolts	
aVR. R" Amp	Integer	Continuous	microvolts	
aVR. S" Amp	Integer	Continuous	microvolts	
aVR. ST Amp	Integer	Continuous	microvolts	
aVR. STT28 Amp	Integer	Continuous	microvolts	
aVR. STT38 Amp	Integer	Continuous	microvolts	
aVR. T+ Amp	Integer	Continuous	microvolts	
aVR. T- Amp	Integer	Continuous	microvolts	
aVR. QRS Area	Integer	Continuous	uvolt-msec/20	
aVR. P Area	Integer	Continuous	uvolt-msec/20	
aVR. T Area	Integer	Continuous	uvolt-msec/20	
aVR. P Morph	Integer	Categorical	N/A	P morphology (INT: -2 to +2)
aVR. T Morph	Integer	Categorical	N/A	T morphology (INT: -2 to +2)

aVR. R Notch	Integer	Continuous	N/A	R wave notches
aVR. DeltaConf	Integer	Continuous	percent	Delta wave confidence (INT: 0 to 100)
aVR. ST Slope	Integer	Continuous	degrees	ST slope (INT: - 90 to + 90)
aVR. QT Int	Integer	Continuous	msec	QT interval
aVL. P Ons	Integer	Continuous	msec	
aVL. P Dur	Integer	Continuous	msec	
aVL. QRS Ons	Integer	Continuous	msec	
aVL. QRS Dur	Integer	Continuous	msec	
aVL. ST80 Amp	Integer	Continuous	microvolts	
aVL. Q Dur	Integer	Continuous	msec	
aVL. R Dur	Integer	Continuous	msec	
aVL. S Dur	Integer	Continuous	msec	
aVL. R' Dur	Integer	Continuous	msec	
aVL. S' Dur	Integer	Continuous	msec	
aVL. R" Dur	Integer	Continuous	msec	
aVL. S" Dur	Integer	Continuous	msec	
aVL. ST Dur	Integer	Continuous	msec	
aVL. T Ons	Integer	Continuous	msec	
aVL. T Dur	Integer	Continuous	msec	
aVL. P+ Dur	Integer	Continuous	msec	
aVL. T+ Dur	Integer	Continuous	msec	
aVL. QRS IntD	Integer	Continuous	msec	
aVL. P+ Amp	Integer	Continuous	microvolts	
aVL. P- Amp	Integer	Continuous	microvolts	
aVL. P2P Amp	Integer	Continuous	microvolts	
aVL. R1 Amp	Integer	Continuous	microvolts	
aVL. Q Amp	Integer	Continuous	microvolts	
aVL. R Amp	Integer	Continuous	microvolts	
aVL. S Amp	Integer	Continuous	microvolts	
aVL. R' Amp	Integer	Continuous	microvolts	
aVL. S' Amp	Integer	Continuous	microvolts	
aVL. R" Amp	Integer	Continuous	microvolts	
aVL. S" Amp	Integer	Continuous	microvolts	
aVL. ST Amp	Integer	Continuous	microvolts	
aVL. STT28 Amp	Integer	Continuous	microvolts	
aVL. STT38 Amp	Integer	Continuous	microvolts	
aVL. T+ Amp	Integer	Continuous	microvolts	
aVL. T- Amp	Integer	Continuous	microvolts	
aVL. QRS Area	Integer	Continuous	uvolt-msec/20	
aVL. P Area	Integer	Continuous	uvolt-msec/20	
aVL. T Area	Integer	Continuous	uvolt-msec/20	
aVL. P Morph	Integer	Categorical	N/A	P morphology (INT: -2 to +2)
aVL. T Morph	Integer	Categorical	N/A	T morphology (INT: -2 to +2)
aVL. R Notch	Integer	Continuous	N/A	R wave notches
aVL. DeltaConf	Integer	Continuous	percent	Delta wave confidence (INT: 0 to 100)
aVL. ST Slope	Integer	Continuous	degrees	ST slope (INT: - 90 to + 90)
aVL. QT Int	Integer	Continuous	msec	QT interval
aVF. P Ons	Integer	Continuous	msec	
aVF. P Dur	Integer	Continuous	msec	

aVF. QRS Ons	Integer	Continuous	msec	
aVF. QRS Dur	Integer	Continuous	msec	
aVF. ST80 Amp	Integer	Continuous	microvolts	
aVF. Q Dur	Integer	Continuous	msec	
aVF. R Dur	Integer	Continuous	msec	
aVF. S Dur	Integer	Continuous	msec	
aVF. R' Dur	Integer	Continuous	msec	
aVF. S' Dur	Integer	Continuous	msec	
aVF. R" Dur	Integer	Continuous	msec	
aVF. S" Dur	Integer	Continuous	msec	
aVF. ST Dur	Integer	Continuous	msec	
aVF. T Ons	Integer	Continuous	msec	
aVF. T Dur	Integer	Continuous	msec	
aVF. P+ Dur	Integer	Continuous	msec	
aVF. T+ Dur	Integer	Continuous	msec	
aVF. QRS IntD	Integer	Continuous	msec	
aVF. P+ Amp	Integer	Continuous	microvolts	
aVF. P- Amp	Integer	Continuous	microvolts	
aVF. P2P Amp	Integer	Continuous	microvolts	
aVF. R1 Amp	Integer	Continuous	microvolts	
aVF. Q Amp	Integer	Continuous	microvolts	
aVF. R Amp	Integer	Continuous	microvolts	
aVF. S Amp	Integer	Continuous	microvolts	
aVF. R' Amp	Integer	Continuous	microvolts	
aVF. S' Amp	Integer	Continuous	microvolts	
aVF. R" Amp	Integer	Continuous	microvolts	
aVF. S" Amp	Integer	Continuous	microvolts	
aVF. ST Amp	Integer	Continuous	microvolts	
aVF. STT28 Amp	Integer	Continuous	microvolts	
aVF. STT38 Amp	Integer	Continuous	microvolts	
aVF. T+ Amp	Integer	Continuous	microvolts	
aVF. T- Amp	Integer	Continuous	microvolts	
aVF. QRS Area	Integer	Continuous	uvolt-msec/20	
aVF. P Area	Integer	Continuous	uvolt-msec/20	
aVF. T Area	Integer	Continuous	uvolt-msec/20	
aVF. P Morph	Integer	Categorical	N/A	P morphology (INT: -2 to +2)
aVF. T Morph	Integer	Categorical	N/A	T morphology (INT: -2 to +2)
aVF. R Notch	Integer	Continuous	N/A	R wave notches
aVF. DeltaConf	Integer	Continuous	percent	Delta wave confidence (INT: 0 to 100)
aVF. ST Slope	Integer	Continuous	degrees	ST slope (INT: - 90 to + 90)
aVF. QT Int	Integer	Continuous	msec	QT interval
V1. P Ons	Integer	Continuous	msec	
V1. P Dur	Integer	Continuous	msec	
V1. QRS Ons	Integer	Continuous	msec	
V1. QRS Dur	Integer	Continuous	msec	
V1. ST80 Amp	Integer	Continuous	microvolts	
V1. Q Dur	Integer	Continuous	msec	
V1. R Dur	Integer	Continuous	msec	
V1. S Dur	Integer	Continuous	msec	

V1. R' Dur	Integer	Continuous	msec	
V1. S' Dur	Integer	Continuous	msec	
V1. R" Dur	Integer	Continuous	msec	
V1. S" Dur	Integer	Continuous	msec	
V1. ST Dur	Integer	Continuous	msec	
V1. T Ons	Integer	Continuous	msec	
V1. T Dur	Integer	Continuous	msec	
V1. P+ Dur	Integer	Continuous	msec	
V1. T+ Dur	Integer	Continuous	msec	
V1. QRS IntD	Integer	Continuous	msec	
V1. P+ Amp	Integer	Continuous	microvolts	
V1. P- Amp	Integer	Continuous	microvolts	
V1. P2P Amp	Integer	Continuous	microvolts	
V1. R1 Amp	Integer	Continuous	microvolts	
V1. Q Amp	Integer	Continuous	microvolts	
V1. R Amp	Integer	Continuous	microvolts	
V1. S Amp	Integer	Continuous	microvolts	
V1. R' Amp	Integer	Continuous	microvolts	
V1. S' Amp	Integer	Continuous	microvolts	
V1. R" Amp	Integer	Continuous	microvolts	
V1. S" Amp	Integer	Continuous	microvolts	
V1. ST Amp	Integer	Continuous	microvolts	
V1. STT28 Amp	Integer	Continuous	microvolts	
V1. STT38 Amp	Integer	Continuous	microvolts	
V1. T+ Amp	Integer	Continuous	microvolts	
V1. T- Amp	Integer	Continuous	microvolts	
V1. QRS Area	Integer	Continuous	uvolt-msec/20	
V1. P Area	Integer	Continuous	uvolt-msec/20	
V1. T Area	Integer	Continuous	uvolt-msec/20	
V1. P Morph	Integer	Categorical	N/A	P morphology (INT: -2 to +2)
V1. T Morph	Integer	Categorical	N/A	T morphology (INT: -2 to +2)
V1. R Notch	Integer	Continuous	N/A	R wave notches
V1. DeltaConf	Integer	Continuous	percent	Delta wave confidence (INT: 0 to 100)
V1. ST Slope	Integer	Continuous	degrees	ST slope (INT: - 90 to + 90)
V1. QT Int	Integer	Continuous	msec	QT interval
V2. P Ons	Integer	Continuous	msec	
V2. P Dur	Integer	Continuous	msec	
V2. QRS Ons	Integer	Continuous	msec	
V2. QRS Dur	Integer	Continuous	msec	
V2. ST80 Amp	Integer	Continuous	microvolts	
V2. Q Dur	Integer	Continuous	msec	
V2. R Dur	Integer	Continuous	msec	
V2. S Dur	Integer	Continuous	msec	
V2. R' Dur	Integer	Continuous	msec	
V2. S' Dur	Integer	Continuous	msec	
V2. R" Dur	Integer	Continuous	msec	
V2. S" Dur	Integer	Continuous	msec	
V2. ST Dur	Integer	Continuous	msec	
V2. T Ons	Integer	Continuous	msec	

V2. T Dur	Integer	Continuous	msec	
V2. P+ Dur	Integer	Continuous	msec	
V2. T+ Dur	Integer	Continuous	msec	
V2. QRS IntD	Integer	Continuous	msec	
V2. P+ Amp	Integer	Continuous	microvolts	
V2. P- Amp	Integer	Continuous	microvolts	
V2. P2P Amp	Integer	Continuous	microvolts	
V2. R1 Amp	Integer	Continuous	microvolts	
V2. Q Amp	Integer	Continuous	microvolts	
V2. R Amp	Integer	Continuous	microvolts	
V2. S Amp	Integer	Continuous	microvolts	
V2. R' Amp	Integer	Continuous	microvolts	
V2. S' Amp	Integer	Continuous	microvolts	
V2. R" Amp	Integer	Continuous	microvolts	
V2. S" Amp	Integer	Continuous	microvolts	
V2. ST Amp	Integer	Continuous	microvolts	
V2. STT28 Amp	Integer	Continuous	microvolts	
V2. STT38 Amp	Integer	Continuous	microvolts	
V2. T+ Amp	Integer	Continuous	microvolts	
V2. T- Amp	Integer	Continuous	microvolts	
V2. QRS Area	Integer	Continuous	uvolt-msec/20	
V2. P Area	Integer	Continuous	uvolt-msec/20	
V2. T Area	Integer	Continuous	uvolt-msec/20	
V2. P Morph	Integer	Categorical	N/A	P morphology (INT: -2 to +2)
V2. T Morph	Integer	Categorical	N/A	T morphology (INT: -2 to +2)
V2. R Notch	Integer	Continuous	N/A	R wave notches
V2. DeltaConf	Integer	Continuous	percent	Delta wave confidence (INT: 0 to 100)
V2. ST Slope	Integer	Continuous	degrees	ST slope (INT: - 90 to + 90)
V2. QT Int	Integer	Continuous	msec	QT interval
V3. P Ons	Integer	Continuous	msec	
V3. P Dur	Integer	Continuous	msec	
V3. QRS Ons	Integer	Continuous	msec	
V3. QRS Dur	Integer	Continuous	msec	
V3. ST80 Amp	Integer	Continuous	microvolts	
V3. Q Dur	Integer	Continuous	msec	
V3. R Dur	Integer	Continuous	msec	
V3. S Dur	Integer	Continuous	msec	
V3. R' Dur	Integer	Continuous	msec	
V3. S' Dur	Integer	Continuous	msec	
V3. R" Dur	Integer	Continuous	msec	
V3. S" Dur	Integer	Continuous	msec	
V3. ST Dur	Integer	Continuous	msec	
V3. T Ons	Integer	Continuous	msec	
V3. T Dur	Integer	Continuous	msec	
V3. P+ Dur	Integer	Continuous	msec	
V3. T+ Dur	Integer	Continuous	msec	
V3. QRS IntD	Integer	Continuous	msec	
V3. P+ Amp	Integer	Continuous	microvolts	
V3. P- Amp	Integer	Continuous	microvolts	

V3. P2P Amp	Integer	Continuous	microvolts	
V3. R1 Amp	Integer	Continuous	microvolts	
V3. Q Amp	Integer	Continuous	microvolts	
V3. R Amp	Integer	Continuous	microvolts	
V3. S Amp	Integer	Continuous	microvolts	
V3. R' Amp	Integer	Continuous	microvolts	
V3. S' Amp	Integer	Continuous	microvolts	
V3. R" Amp	Integer	Continuous	microvolts	
V3. S" Amp	Integer	Continuous	microvolts	
V3. ST Amp	Integer	Continuous	microvolts	
V3. STT28 Amp	Integer	Continuous	microvolts	
V3. STT38 Amp	Integer	Continuous	microvolts	
V3. T+ Amp	Integer	Continuous	microvolts	
V3. T- Amp	Integer	Continuous	microvolts	
V3. QRS Area	Integer	Continuous	uvolt-msec/20	
V3. P Area	Integer	Continuous	uvolt-msec/20	
V3. T Area	Integer	Continuous	uvolt-msec/20	
V3. P Morph	Integer	Categorical	N/A	P morphology (INT: -2 to +2)
V3. T Morph	Integer	Categorical	N/A	T morphology (INT: -2 to +2)
V3. R Notch	Integer	Continuous	N/A	R wave notches
V3. DeltaConf	Integer	Continuous	percent	Delta wave confidence (INT: 0 to 100)
V3. ST Slope	Integer	Continuous	degrees	ST slope (INT: - 90 to + 90)
V3. QT Int	Integer	Continuous	msec	QT interval
V4. P Ons	Integer	Continuous	msec	
V4. P Dur	Integer	Continuous	msec	
V4. QRS Ons	Integer	Continuous	msec	
V4. QRS Dur	Integer	Continuous	msec	
V4. ST80 Amp	Integer	Continuous	microvolts	
V4. Q Dur	Integer	Continuous	msec	
V4. R Dur	Integer	Continuous	msec	
V4. S Dur	Integer	Continuous	msec	
V4. R' Dur	Integer	Continuous	msec	
V4. S' Dur	Integer	Continuous	msec	
V4. R" Dur	Integer	Continuous	msec	
V4. S" Dur	Integer	Continuous	msec	
V4. ST Dur	Integer	Continuous	msec	
V4. T Ons	Integer	Continuous	msec	
V4. T Dur	Integer	Continuous	msec	
V4. P+ Dur	Integer	Continuous	msec	
V4. T+ Dur	Integer	Continuous	msec	
V4. QRS IntD	Integer	Continuous	msec	
V4. P+ Amp	Integer	Continuous	microvolts	
V4. P- Amp	Integer	Continuous	microvolts	
V4. P2P Amp	Integer	Continuous	microvolts	
V4. R1 Amp	Integer	Continuous	microvolts	
V4. Q Amp	Integer	Continuous	microvolts	
V4. R Amp	Integer	Continuous	microvolts	
V4. S Amp	Integer	Continuous	microvolts	
V4. R' Amp	Integer	Continuous	microvolts	

V4. S' Amp	Integer	Continuous	microvolts	
V4. R" Amp	Integer	Continuous	microvolts	
V4. S" Amp	Integer	Continuous	microvolts	
V4. ST Amp	Integer	Continuous	microvolts	
V4. STT28 Amp	Integer	Continuous	microvolts	
V4. STT38 Amp	Integer	Continuous	microvolts	
V4. T+ Amp	Integer	Continuous	microvolts	
V4. T- Amp	Integer	Continuous	microvolts	
V4. QRS Area	Integer	Continuous	uvolt-msec/20	
V4. P Area	Integer	Continuous	uvolt-msec/20	
V4. T Area	Integer	Continuous	uvolt-msec/20	
V4. P Morph	Integer	Categorical	N/A	P morphology (INT: -2 to +2)
V4. T Morph	Integer	Categorical	N/A	T morphology (INT: -2 to +2)
V4. R Notch	Integer	Continuous	N/A	R wave notches
V4. DeltaConf	Integer	Continuous	percent	Delta wave confidence (INT: 0 to 100)
V4. ST Slope	Integer	Continuous	degrees	ST slope (INT: - 90 to + 90)
V4. QT Int	Integer	Continuous	msec	QT interval
V5. P Ons	Integer	Continuous	msec	
V5. P Dur	Integer	Continuous	msec	
V5. QRS Ons	Integer	Continuous	msec	
V5. QRS Dur	Integer	Continuous	msec	
V5. ST80 Amp	Integer	Continuous	microvolts	
V5. Q Dur	Integer	Continuous	msec	
V5. R Dur	Integer	Continuous	msec	
V5. S Dur	Integer	Continuous	msec	
V5. R' Dur	Integer	Continuous	msec	
V5. S' Dur	Integer	Continuous	msec	
V5. R" Dur	Integer	Continuous	msec	
V5. S" Dur	Integer	Continuous	msec	
V5. ST Dur	Integer	Continuous	msec	
V5. T Ons	Integer	Continuous	msec	
V5. T Dur	Integer	Continuous	msec	
V5. P+ Dur	Integer	Continuous	msec	
V5. T+ Dur	Integer	Continuous	msec	
V5. QRS IntD	Integer	Continuous	msec	
V5. P+ Amp	Integer	Continuous	microvolts	
V5. P- Amp	Integer	Continuous	microvolts	
V5. P2P Amp	Integer	Continuous	microvolts	
V5. R1 Amp	Integer	Continuous	microvolts	
V5. Q Amp	Integer	Continuous	microvolts	
V5. R Amp	Integer	Continuous	microvolts	
V5. S Amp	Integer	Continuous	microvolts	
V5. R' Amp	Integer	Continuous	microvolts	
V5. S' Amp	Integer	Continuous	microvolts	
V5. R" Amp	Integer	Continuous	microvolts	
V5. S" Amp	Integer	Continuous	microvolts	
V5. ST Amp	Integer	Continuous	microvolts	
V5. STT28 Amp	Integer	Continuous	microvolts	
V5. STT38 Amp	Integer	Continuous	microvolts	

V5. T+ Amp	Integer	Continuous	microvolts	
V5. T- Amp	Integer	Continuous	microvolts	
V5. QRS Area	Integer	Continuous	uvolt-msec/20	
V5. P Area	Integer	Continuous	uvolt-msec/20	
V5. T Area	Integer	Continuous	uvolt-msec/20	
V5. P Morph	Integer	Categorical	N/A	P morphology (INT: -2 to +2)
V5. T Morph	Integer	Categorical	N/A	T morphology (INT: -2 to +2)
V5. R Notch	Integer	Continuous	N/A	R wave notches
V5. DeltaConf	Integer	Continuous	percent	Delta wave confidence (INT: 0 to 100)
V5. ST Slope	Integer	Continuous	degrees	ST slope (INT: - 90 to + 90)
V5. QT Int	Integer	Continuous	msec	QT interval
V6. P Ons	Integer	Continuous	msec	
V6. P Dur	Integer	Continuous	msec	
V6. QRS Ons	Integer	Continuous	msec	
V6. QRS Dur	Integer	Continuous	msec	
V6. ST80 Amp	Integer	Continuous	microvolts	
V6. Q Dur	Integer	Continuous	msec	
V6. R Dur	Integer	Continuous	msec	
V6. S Dur	Integer	Continuous	msec	
V6. R' Dur	Integer	Continuous	msec	
V6. S' Dur	Integer	Continuous	msec	
V6. R" Dur	Integer	Continuous	msec	
V6. S" Dur	Integer	Continuous	msec	
V6. ST Dur	Integer	Continuous	msec	
V6. T Ons	Integer	Continuous	msec	
V6. T Dur	Integer	Continuous	msec	
V6. P+ Dur	Integer	Continuous	msec	
V6. T+ Dur	Integer	Continuous	msec	
V6. QRS IntD	Integer	Continuous	msec	
V6. P+ Amp	Integer	Continuous	microvolts	
V6. P- Amp	Integer	Continuous	microvolts	
V6. P2P Amp	Integer	Continuous	microvolts	
V6. R1 Amp	Integer	Continuous	microvolts	
V6. Q Amp	Integer	Continuous	microvolts	
V6. R Amp	Integer	Continuous	microvolts	
V6. S Amp	Integer	Continuous	microvolts	
V6. R' Amp	Integer	Continuous	microvolts	
V6. S' Amp	Integer	Continuous	microvolts	
V6. R" Amp	Integer	Continuous	microvolts	
V6. S" Amp	Integer	Continuous	microvolts	
V6. ST Amp	Integer	Continuous	microvolts	
V6. STT28 Amp	Integer	Continuous	microvolts	
V6. STT38 Amp	Integer	Continuous	microvolts	
V6. T+ Amp	Integer	Continuous	microvolts	
V6. T- Amp	Integer	Continuous	microvolts	
V6. QRS Area	Integer	Continuous	uvolt-msec/20	
V6. P Area	Integer	Continuous	uvolt-msec/20	
V6. T Area	Integer	Continuous	uvolt-msec/20	
V6. P Morph	Integer	Categorical	N/A	P morphology (INT: -2 to +2)

V6. T Morph	Integer	Categorical	N/A	T morphology (INT: -2 to +2)
V6. R Notch	Integer	Continuous	N/A	R wave notches
V6. DeltaConf	Integer	Continuous	percent	Delta wave confidence (INT: 0 to 100)
V6. ST Slope	Integer	Continuous	degrees	ST slope (INT: - 90 to + 90)
V6. QT Int	Integer	Continuous	msec	QT interval
