# Supplementary material for: Use of the energy waveform electrocardiogram to detect subclinical left ventricular dysfunction in patients with type 2 diabetes mellitus
Source: Cardiovasc Diabetol. 2024 Mar 6;23:91. doi: 10.1186/s12933-024-02141-1 (PMC10918872; doi:10.1186/s12933-024-02141-1)
Supplement: Supplementary file 2 — Additional file 2. [file 12933_2024_2141_MOESM2_ESM.docx]

Supplementary Figure 2: The SHAP interpretation plot for the machine learning model for SBHF screening.


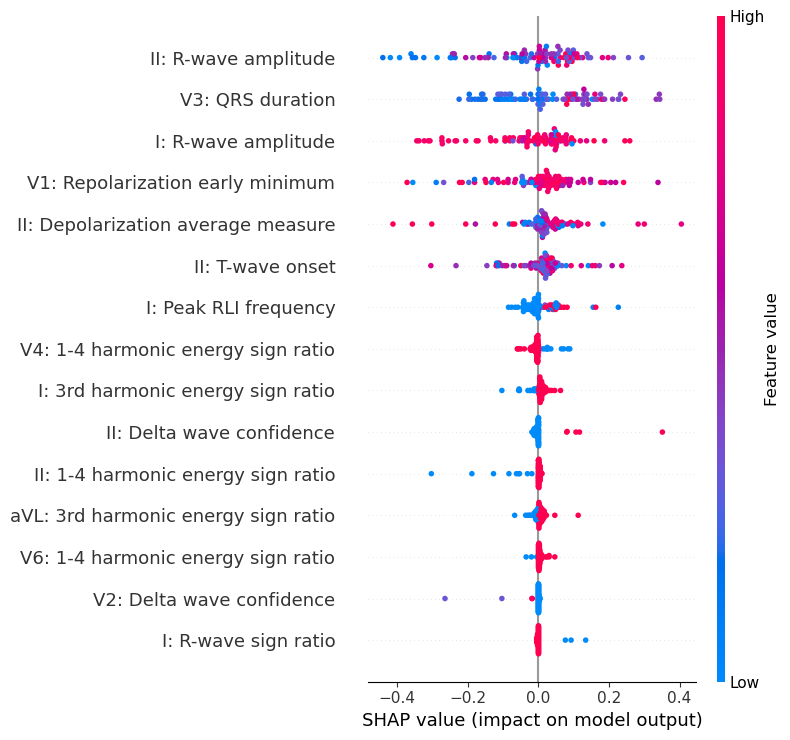


[SHAP: Shapley Additive Explanations; SBHF: Stage B Heart Failure. RLI: Repolarization late minimum. SHAP is a feature importance plot that shows the average absolute SHAP values for each predictor in descending order of importance. The amplitude of R-wave in lead II was shown to be the feature with greatest impact in SBHF screening, with higher value indicating greater risk of SBHF. Similar direction of association was shown in features such as duration of QRS interval and frequency of peak RLI. On the other hand, the sign ratio of 1-4 harmonic energy to the total energy in lead V4 was an example of opposite direction with lower value indicating higher risk of SBHF. Lastly, despite being selected as one of the key features for SBHF screening, features such as amplitude of R-wave in lead I and T-wave onset in lead II showed a mixed pattern and therefore, making it difficult to interpret on the surface value.]

Supplementary Figure 3: SHAP interpretation plot for the machine learning model screening for diastolic dysfunction.


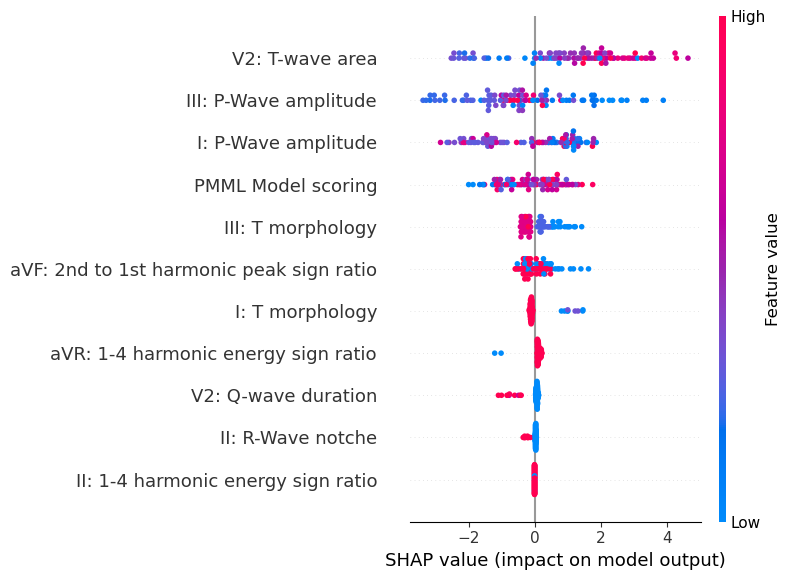


[SHAP: Shapley Additive Explanations; PMML: Predictive Model Markup Language. T-wave area in lead V2 was shown to be the most impactful feature with higher value indicating higher risk of diastolic dysfunction. The PMML model scoring followed a similar direction in association. On the other hand, T-wave morphology in lead I and III were examples of opposite direction with lower values indicating higher risk of diastolic dysfunction. Lastly, P-wave amplitude in lead I and III showed a mixed pattern in screening for diastolic dysfunction.]

Supplementary Figure 4: SHAP interpretation plot for the machine learning model screening for reduced GLS.


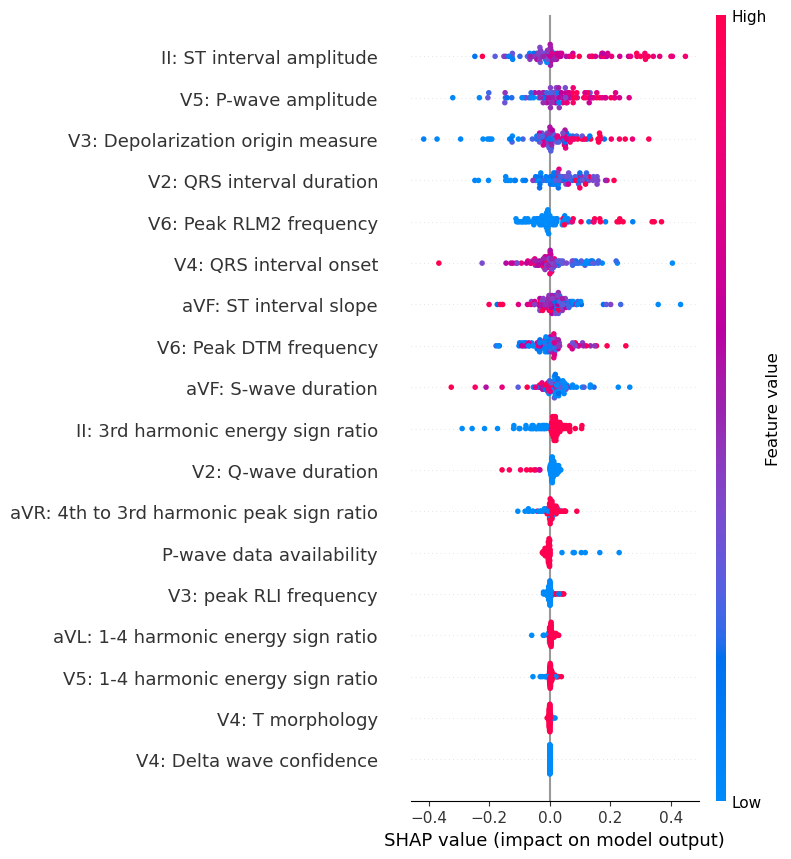


[SHAP: Shapley Additive Explanations; GLS: global longitudinal strain; RLM: repolarization late maximum; RLI: repolarization late minimum. Amplitude of ST interval in lead II was the most impactful feature, with higher value indicating greater risk of reduced GLS, similar to features such as the P-wave amplitude in lead V5 and depolarization origin measure in lead V3. Duration of Q wave in lead V2 showed an opposite direction with lower values indicating higher risk of reduced GLS.]

Supplementary Figure 5: SHAP interpretation plot for the machine learning model screening for LVH.


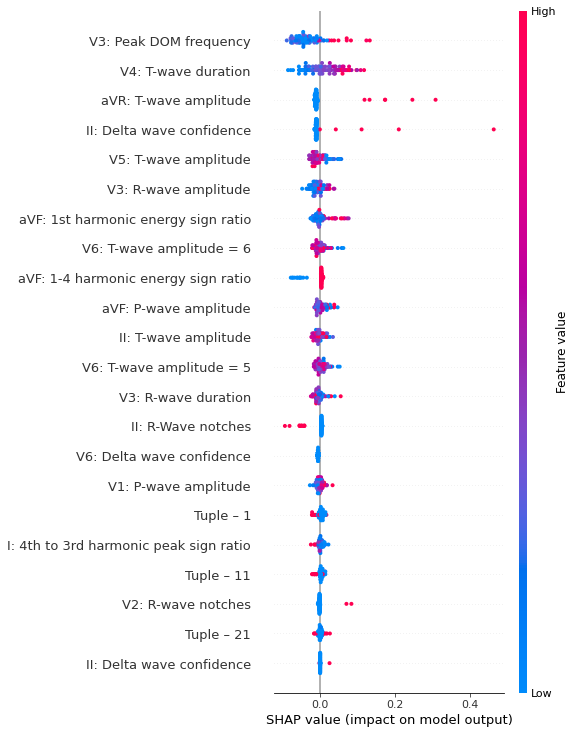


[SHAP: Shapley Additive Explanations; LVH: left ventricular hypertrophy; DOM: Depolarization origin measure. Of the 22 features selected to build the model of LVH screening, the peak frequency for DOM in lead V3 showed the highest impact in the screening model. The duration of T-wave in lead V4 and amplitude of T-wave in lead aVR followed a similar direction of association with peak DOM frequency in the screening of LVH.]
